# Fast and flexible profiling of chromatin accessibility and total RNA expression in single nuclei using Microwell-seq3

**DOI:** 10.1038/s41421-023-00642-z

**Published:** 2024-03-26

**Authors:** Fang Ye, Shuang Zhang, Yuting Fu, Lei Yang, Guodong Zhang, Yijun Wu, Jun Pan, Haide Chen, Xinru Wang, Lifeng Ma, Haofu Niu, Mengmeng Jiang, Tingyue Zhang, Danmei Jia, Jingjing Wang, Yongcheng Wang, Xiaoping Han, Guoji Guo

**Affiliations:** 1 Bone Marrow Transplantation Center of the First Affiliated Hospital, and Center for Stem Cell and Regenerative Medicine, Zhejiang University School of Medicine, Hangzhou, Zhejiang China; 2 Liangzhu Laboratory, Zhejiang University, Hangzhou, Zhejiang China; 3 Department of Thyroid Surgery, The First Affiliated Hospital, Zhejiang University School of Medicine, Hangzhou, Zhejiang China; 4 Zhejiang University–University of Edinburgh Institute, Zhejiang University School of Medicine, Zhejiang University, Hangzhou, Zhejiang China; 5 Zhejiang Provincial Key Lab for Tissue Engineering and Regenerative Medicine, Dr. Li Dak Sum & Yip Yio Chin Center for Stem Cell and Regenerative Medicine, Hangzhou, Zhejiang China; 6 Institute of Hematology, Zhejiang University, Hangzhou, Zhejiang China

**Keywords:** Chromatin analysis, Gene expression profiling

## Abstract

Single cell chromatin accessibility profiling and transcriptome sequencing are the most widely used technologies for single-cell genomics. Here, we present Microwell-seq3, a high-throughput and facile platform for high-sensitivity single-nucleus chromatin accessibility or full-length transcriptome profiling. The method combines a preindexing strategy and a penetrable chip-in-a-tube for single nucleus loading and DNA amplification and therefore does not require specialized equipment. We used Microwell-seq3 to profile chromatin accessibility in more than 200,000 single nuclei and the full-length transcriptome in ~50,000 nuclei from multiple adult mouse tissues. Compared with the existing polyadenylated transcript capture methods, integrative analysis of cell type-specific regulatory elements and total RNA expression uncovered comprehensive cell type heterogeneity in the brain. Gene regulatory networks based on chromatin accessibility profiling provided an improved cell type communication model. Finally, we demonstrated that Microwell-seq3 can identify malignant cells and their specific regulons in spontaneous lung tumors of aged mice. We envision a broad application of Microwell-seq3 in many areas of research.

## Introduction

In the past decades, technical advances in single-cell omics have led to the evolution of knowledge regarding aspects of cellular and molecular biology in health and disease^1–3^. Currently, single-cell omics have covered the genome^4,5^, transcriptome^6–9^, chromatin accessibility^10–12^, proteome^13,14^ and other layers of epigenomes^15–17^. Plate-based methods have initially shaped the single-cell transcriptomic field^6,7,18^. Later, different approaches for single-cell barcoding, including droplet-based microfluidic chips^19–21^, microwells^22,23^, and pool-split strategies^24–26^ have significantly increased the throughput of single-cell sequencing^27^. However, commercialized platforms (e.g. 10X Genomics) or no-instrument strategies (e.g. Parse Biosciences, Fluent Biosciences) are still costly. In addition, sensitivity remains a problem for most single-cell assays. For example, many high-throughput oligo-dT-based methods capture only the 3′ end of polyadenylated transcripts without a full-length coverage. Common methods for high-throughput assay for transposase-accessible chromatin sequencing (ATAC-seq) usually use fixed nuclei, which may result in a relatively low transcription start site (TSS) enrichment score^28^.

To get around the limitation of 3′-end bias in single-cell RNA-seq, full-length single-cell transcriptome sequencing methods were developed to detect total RNA with high sensitivity^29–32^. Plate-based full-length whole-transcriptome methods are restricted by throughput and sorting instruments^29,31–35^. The characteristics of microfluidic systems also increase the difficulty in preparing library in some full-length methods^30,32^. In order to provide a low-cost method for single-cell sequencing, we previously developed Microwell-seq^22^ and Microwell-seq 2.0^36^ for 3′ end transcriptome profiling using off-the-shelf reagents. However, the complex loading workflow limits the application of current protocols. Here, we present Microwell-seq3, which is easy loading and appropriate for both single-nucleus RNA-seq (snRNA-seq) and snATAC-seq. Microwell-seq3 adopts random priming to capture non-polyadenylated and polyadenylated transcripts. For high-throughput snATAC-seq, Microwell-seq3 optimizes the tagmentation step with fresh or frozen nuclei to obtain high-quality TSS enrichment. Our method combines a combinatorial indexing strategy and a chip-in-a-tube design without the need for any specialized equipment. We applied Microwell-seq3 to construct the transcriptomic and chromatin accessibility landscapes of multiple adult mouse tissues. Finally, we characterized the copy number variations (CNVs) and regulon networks in spontaneous lung tumors from aged mice to identify the molecular state of malignant cells.

## Results

### Microwell-seq3 workflow

**Fig. 1:**
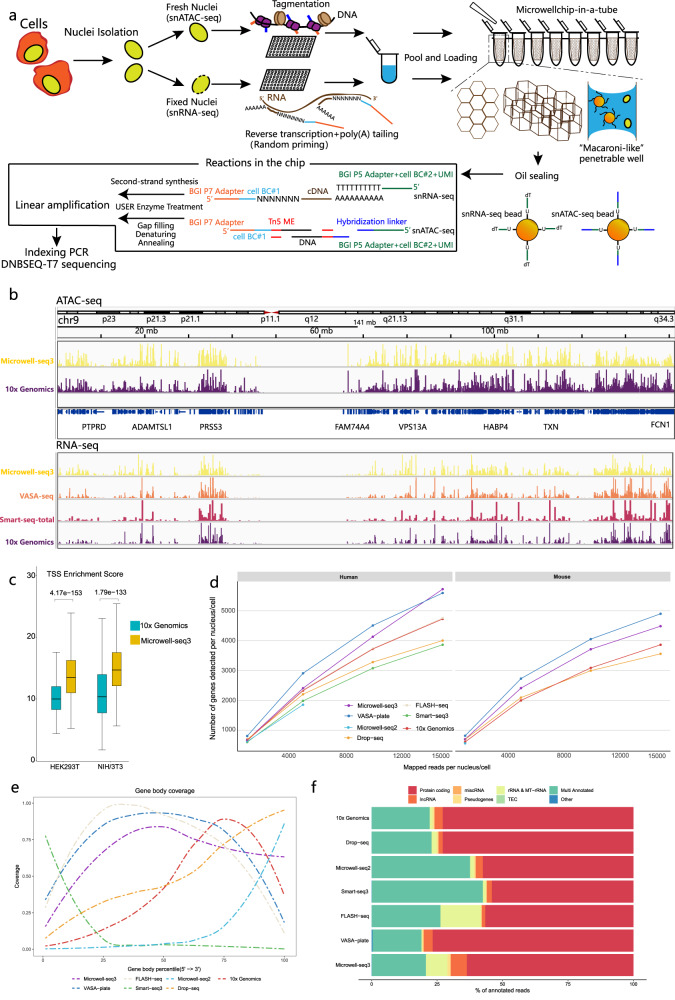
Overview of the microwell-seq3 workflow. **a** Schematic view of the Microwell-seq3 workflow. Single nuclei are extracted from fresh or frozen tissues. DNA or RNA in nuclei is tagged with the first part of the cell barcode (BC#1) in multiple 96-well plates. Labeled nuclei and barcoded magnetic beads are pooled and evenly loaded into Microwell chips. Oligos are released from the beads and linear preamplification is performed in the chips. Preamplified DNA or RNA fragments are collected from the chips for final library preparation and sequencing. **b** Representative University of California Santa Cruz (UCSC) Genome Browser view of ATAC-seq and RNA-seq signal tracks (HEK293T cells) from Microwell-seq3, 10X Genomics, VASA-seq and Smart-seq-total data. **c** Distribution of TSS enrichment scores from Microwell-seq3 and 10X Genomics ATAC-seq data in the indicated cell lines, reads (fragments) are down sampled to 2000 fragments per nucleus. The statistical test used is a two-sided Student’s *t*-test. **d** Number of the detected annotated genes in human and mouse cell lines (HEK293T, mouse NIH/3T3 and embryonic stem cells (mESCs)) in each method is plotted against the number of unique mapped reads per cell in different down-sampling thresholds. All the reads are remapped with the same pipeline after adapter filtering and trimming. Data of mESCs were generated by VASA-seq. **e** Comparison of mean ± standard deviation (SD) gene body coverage in protein-coding genes in cell lines across the different methods. Microwell-seq3, FLASH-seq and VASA-seq show even coverage across the length of the genes. Other methods show bias toward the 5′ and 3′ ends of transcripts, respectively. Reads in each method are trimmed to 500,000 per sample. **f** Proportions of the reads mapped to all annotated genes for each biotype in cell lines across the different methods. Microwell-seq3 detects proportionally higher levels of lncRNAs. Reads in each method are trimmed to 5000 per cell.

Microwell-seq3 utilized in situ preindexing of molecules after nucleus isolation (Fig. 1a). The nuclei can be split into different plates for either RNA-seq or ATAC-seq. In the RNA-seq workflow, nuclei were partitioned into multiple 96-well plates, and reverse transcription (RT) was performed with barcoded random primers to introduce P7 sequencing adapter and the first part of the cell barcode (BC#1) (Supplementary Table S1). After exonuclease I treatment of the redundant oligos and poly(A) tailing using terminal deoxynucleotidyl transferase, nuclei were pooled for chip loading. In the ATAC-seq workflow, Tn5 transposase embedded with the first part of the cell barcode (BC#1) and P7 sequencing adapters was added into multiple 96-well plates to tag open regions in chromatin. Then, treated nuclei (from RNA-seq and ATAC-seq workflows) and magnetic beads with the second part of the cell barcode (BC#2) were fully mixed and pipetted into microwell chips. The beads used for snRNA-seq contained a poly(T) tail, unique molecular identifiers (UMIs) and P5 sequencing adapters. Alternatively, the beads used for snATAC-seq contained a hybridization linker tail to capture the DNA fragments.

The chip-in-a-tube approach was originally utilized in digital polymerase chain reaction (dPCR) system^37^. In the system presented herein, the 10,000 subdivided “macaroni-like” partitions allow the trapping of multiple nuclei and beads by capillary effect (Supplementary Fig. S1a). Thus, the penetrable wells do not rely on gravity and the Poisson probability distribution, as do other microwell-based methods with a limited cell trap efficiency^22,23,36,38^. Of note, unlike the sealed microfluidic system, the penetrable wells allow multiple rounds of loading for other potential antibody-labeling-based applications in a reagent-saving manner (Supplementary Fig. S1b). In each round of the reaction, the chip can be incubated at the desired temperature. This strategy is motivated by the need to provide the possibility for a multi-step treatment of DNA, RNA and proteins in single cells or nuclei. Oil sealing of the chip surface effectively prevents potential cross-contamination during amplification steps.

In the microwell chip, USER enzyme treatment released the oligos from the beads. Thus, cDNA synthesis (snRNA-seq), DNA gap filling (snATAC-seq) and linear preamplification were integrated into a one-step reaction in the chip. Finally, we recovered all the reaction mixture and discarded the beads. After DNA purification, the second round of amplification was performed to introduce the library index for next generation sequencing (NGS) (detailed Microwell-seq3 protocols are available in Supplementary Methods). The effective microwells with multiple beads and nuclei can be distinguished and decoded through the following data analysis procedure.

### Benchmarking and data quality control of Microwell-seq3

As proof-of-concept, we performed species-mixing experiments with mouse NIH/3T3 cells and human HEK 293 T cells. Benchmarking of our data and external published data generated by other representative methods was performed. The genome-wide read signals around TSSs (ATAC-seq) and transcription termination sites (TTSs) (RNA-seq) demonstrated similar region enrichment profiles among Microwell-seq3, 10X Genomics and other full-length scRNA-seq methods, including VASA-seq^30^ and Smart-seq-total^33^ (Fig. 1b). We observed doublet rates of 0.93% and 1.2% for snRNA-seq and snATAC-seq data, respectively (Supplementary Fig. S1e). Chromatin accessibility profiling with Microwell-seq3 generated a higher TSS score than that obtained with the 10X Genomics (Fig. 1c). Peak annotation^39^ using snATAC-seq fragments demonstrated a typical distribution of ~20% peaks in the promoter region (Supplementary Fig. S1d).

In snRNA-seq, we benchmarked different methods after data remapping and down-sampling. We observed an improved gene detection ability in Microwell-seq3 compared with our previous version and other methods (Fig. 1d). Full length RNA-seq methods (Microwell-seq3, VASA-seq, Smart-seq3^29^, FLASH-seq^35^) demonstrated whole gene body coverage from the 5′ end to the 3′ end of protein-coding sequences, while reads obtained by other methods were enriched at the 3′ end (Fig. 1e). In addition, 9.3% of the detected reads in Microwell-seq3 were annotated as long noncoding RNAs (Fig. 1f; Supplementary Fig. S1c). Both FLASH-seq and Microwell-seq3 detected higher proportions of ribosomal RNAs. The full-length scRNA-seq methods captured different types of short noncoding RNAs (Supplementary Fig. S1f). Only Microwell-seq3 and VASA-seq detected small nuclear RNAs (snRNAs), while VASA-seq detected more miscellaneous RNAs and small nucleolar RNAs.

Paraformaldehyde crosslinking of RNAs and proteins in nuclei blocks reverse transcription of long fragments. We compared the sensitivity between two fixation time periods in a shallow sequencing depth. Slight fixation with a short time resulted a higher gene detection sensitivity (Supplementary Fig. S1g). Moreover, random priming of crosslinked RNA avoided Tn5 tagmentation, which is a time-consuming and costly step in conventional RNA-seq library preparation. Thus, the entire snRNA-seq process of Microwell-seq3 could be achieved by one researcher within 8 h. As for snATAC-seq, the entire library preparation process could be achieved by one researcher within only 6 h with a total cost of $0.06 per cell (for cells passed quality control). Compared with other non-commercial combinatorial indexing methods^40,41^ (e.g. sci-RNA-seq ($0.28 per cell), sci-ATAC-seq ($0.25 per cell), s3-ATAC-seq^42^ ($3.80 per cell)), Microwell-seq3 enabled a more flexible workflow in a time- and cost-saving manner (Supplementary Fig. S1h).

In order to identify the “Cell-containing wells with beads” in the chip (microwells with at least one cell and one bead). We applied a strategy of computing the insertion similarity coefficient (Jaccard index) for overloaded beads in the same microwell (see Materials and methods)^11^. The bead barcodes sharing a noticeable overlap of certain oligonucleotides would be barcodes from two beads in the same well. In snATAC-seq, a diverse library of random oligonucleotides (containing bead capture linker and 14 random nucleotides, 14 N, Supplementary Table S1) were spiked into the cell-bead mixture and loaded into the chip. The calculation of Jaccard index was facilitated on the insertion positions of paired-end reads and refined by spike-in random oligonucleotides (Supplementary Fig. S1i, right). In snRNA-seq, however, the Jaccard index was identified using self-containing random oligonucleotides in the barcoded RT primers and beads (17 random nucleotides, 17 N) without spike-in random oligonucleotides (Supplementary Fig. S1i, left). Bead pairs with certain similarity coefficients were assigned into one “Cell-containing well with beads”. We determined the distribution frequency of the beads in the chip (Supplementary Fig. S1j). Microwells with < 4 beads constituted the majority of the “Cell-containing wells with beads”. We removed the wells with >6 beads. In microwells with more than one nucleus, we further assigned the reads based on the RT cell barcodes or Tn5 adapter barcodes to construct the final matrix files.

### Mapping of the adult mouse cellular landscape using Microwell-seq3

**Fig. 2:**
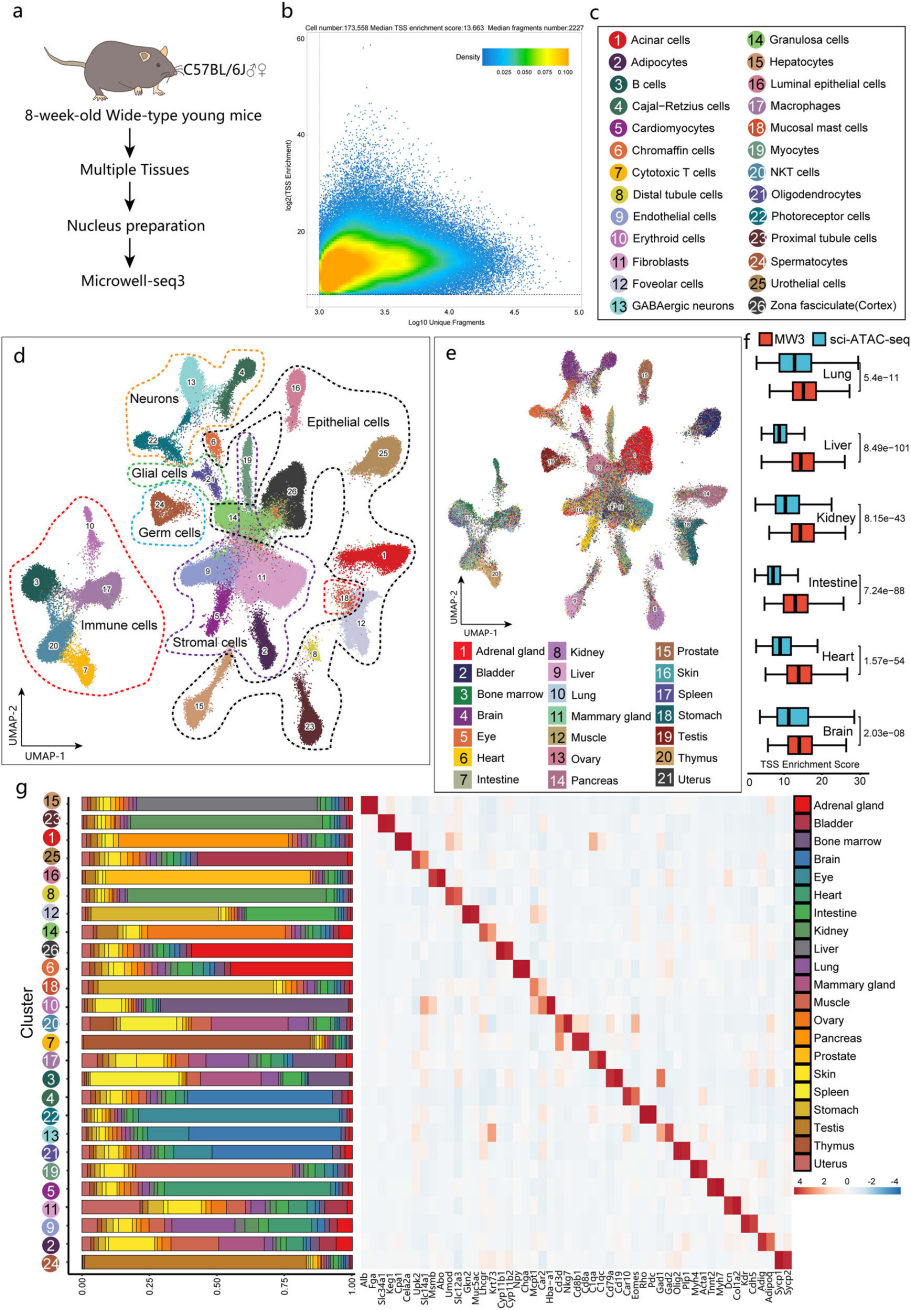
Analysis of chromatin accessibility in multiple mouse tissues. **a** Schematic view of the workflow. **b** Quality control plots generated with ArchR for all cells in the Microwell-seq3 ATAC-seq data from wild-type mice showing the TSS enrichment score and number of unique nuclear DNA fragments per cell. The dot color represents the density (in arbitrary units) of the point in the plot. **c** Annotations of major snATAC-seq clusters in 8-week-old wild-type mice. **d** UMAP plot of all major cell types from 8-week-old wild-type mice in ATAC-seq. **e** UMAP plot of all cell clusters from 8-week-old wild-type mice colored by tissue. **f** Distribution of TSS enrichment scores from Microwell-seq3 and sci-ATAC-seq data in different tissues, reads (fragments) are down sampled to 2000 fragments per nucleus. The statistical test used is a two-sided Student’s *t*-test. **g** Heatmap of differential accessibility relative to the annotated cell type. Significant highly accessible sites in relevant genes are highlighted along the bottom. The proportion of each cluster originating from each tissue is shown alongside the heatmap.

Next, we tested whether Microwell-seq3 can distinguish distinct cell types in a wide range of tissues. To this end, we collected nuclei from multiple adult mouse tissues and used Microwell-seq3 to generate the snATAC-seq and snRNA-seq landscapes (Fig. 2a). After quality control and doublet filtering, we obtained 49,698 cells from the RNA-seq data. The cells were visualized by uniform manifold approximation and projection (UMAP)^43^ (Supplementary Fig. S2a, b). We annotated 27 major cell types from different tissues. High gene expression correlations were observed within the similar cell lineages (Supplementary Fig. S2c). Cell type-specific marker genes enrichment identified a variety of heterogeneous epithelial cells, neurons, endothelial cells and stromal cells (Supplementary Fig. S2d). We analyzed the sensitivity and genome mapping biotype of different tissues (Supplementary Fig. S2e). Although shallow sequencing of a large number of cells resulted in a limited sensitivity, we found a higher detectability in Microwell-seq3 compared with 10X Genomics snRNA-seq data in multiple tissues after data down-sampling (Supplementary Fig. S2f).

We further performed clustering of cell types in each tissue separately. Subclustering analysis of major cell types predicted a total of 164 subclusters in the hierarchy (Supplementary Fig. S3a). Comprehensive tissue-specific epithelial cells (such as hepatocytes, acinar cells, urothelial cells, enterocytes and pit cells), stromal cells (cardiomyocytes, fibroblasts), endothelial cells, neurons, glia and immune cells were identified. Gene expression patterns in Microwell-seq3 RNA-seq data of multiple tissues showed relatively high correlation with the total VASA-seq mouse embryo dataset (both with full-length coverage) (Supplementary Fig. S3b). Comparison between Microwell-seq3 and typical 3′ end gene expression data in 10X Genomics snRNA-seq also showed high correlation in adult mouse tissues (Supplementary Fig. S3b). The kidney demonstrated the highest gene expression correlation with 10X Genomics snRNA-seq data. As one of the most heterogeneous structures, our data unveiled comprehensive cell types in nephron (Supplementary Fig. S3c, d). Inner gene expression correlations in the cell hierarchy showed distinct clustering of cell types (Supplementary Fig. S3e, f). Cross-platform comparison also indicated a credible gene expression similarity between Microwell-seq3 and 10X Genomics (Supplementary Fig. S3g).

Then, we performed clustering of the cells profiled by Microwell-seq3 snATAC-seq, with a total of 173,558 nuclei recovered after quality control (Fig. 2b; Supplementary Fig. S4c). We obtained a median of 2227 unique mapped fragments and a median TSS enrichment score of 13.663. Unsupervised clustering identified 26 major clusters from 21 different tissues (Fig. 2c–e; Supplementary Fig. S4g). We compared the TSS enrichment score between Microwell-seq3 and sci-ATAC-seq (both are pool-split-based methods) (Fig. 2f). An improved performance was observed cross multiple adult mouse tissues in Microwell-seq3. Enrichment of gene regulation markers identified canonical regulators in major cell types (Fig. 2g). To facilitate the identification of the major cell types, we performed label transfer^44^ to integrate Microwell-seq3 ATAC-seq data with Mouse Cell Atlas data (Microwell-seq 1.0)^22^ and Microwell-seq3 RNA-seq data (Supplementary Fig. S4a, b). Integration with Microwell-seq3 RNA-seq data resulted in a better prediction score for parenchymal cells (Supplementary Fig. S4d–f).

### Integrative analyses of an adult mouse brain cell landscape

To make a more systematic comparison of cell types and genes in two modalities, we performed a second round of unsupervised clustering of brain datasets (Supplementary Fig. S5a). There was no significant difference of TSS enrichment score between Microwell-seq3 and 10X Genomics (Supplementary Fig. S5b), whereas down sampled gene expression showed a higher sensitivity in Microwell-seq3 (Supplementary Fig. S5c). Transcription factor (TF) enrichment analysis of the ATAC-seq data clearly distinguished specific open chromatin signals in each cell type (Supplementary Fig. S5e). We profiled the average activity score of the Microwell-seq3 brain ATAC data clusters. Normalized activity scores and gene expression profiles (10X Genomics and Microwell-seq3 RNA-seq data) were compared using Kendall correlation analysis. Gene expression profiles in concordant cell types were correlated well between Microwell-seq3 ATAC and RNA data (Supplementary Fig. S5g). Label transfer of Microwell-seq3 RNA-seq data suggested a high transfer score and identified comprehensive cell types in the ATAC-seq data (Supplementary Fig. S5f, h).

In Microwell-seq3 RNA-seq data, we identified different subtypes of neuron, astrocytes, oligodendrocytes and other nonneuronal cell types with distinct marker gene expression patterns (Supplementary Fig. S5d). After data integration, full-length coverage data in Microwell-seq3 captured more uniquely expressed genes (Supplementary Fig. S6a). Microwell-seq3 detected a slightly lower proportion of protein-coding genes compared to 10X Genomics (Supplementary Fig. S6b). However, snRNAs were only detected in Microwell-seq3. Differential gene expression analysis of overall brain gene signatures identified a series of upregulated genes in the Microwell-seq3 data (Supplementary Fig. S6c). We highlighted non-polyadenylated upregulated genes such as *Ppef2*, *Kcnh6* and *Kcnh8*. Functional enrichment analysis showed that these upregulated genes were associated with potassium and calcium ion sensing and transport functions in neurons (Supplementary Fig. S6d).

We then classified the brain cell types into three major groups (neurons, glia and nonneuronal cells). The upregulated gene module was enriched in neurons (Supplementary Fig. S6e). We performed differential gene expression analysis for all genes across these groups (Supplementary Table S2). Consistent with the above findings, most downregulated genes identified by Microwell-seq3 were localized in mitochondria (*Cox8a*, *Chchd2*) (Supplementary Fig. S6f). Another predominant transcript type among the non-polyadenylated upregulated genes was the fusion transcripts. Both *Schip1* and *Sept5* are associated with neurite extension and neurotransmitter release^45,46^.

Finally, we performed a differential gene expression analysis of common neuron and glia cell types between Microwell-seq3 and 10X Genomics snRNA-seq data (Supplementary Table S3). Interneurons contained the highest number of differentially expressed genes (DEGs) (Supplementary Fig. S6g). Downregulated genes included neuroendocrine secretory pathway gene *Pcsk1n*, microtubule regulator *Rmdn1*, lipid metabolism-associated genes *Apoe* and *Tecr*. Upregulated genes in Microwell-seq3 included neuronal splicing regulator *Rbfox3*, corticogenesis-related transcription factor *Satb2* and presynaptic scaffolding protein-encoding gene *Bsn*. Most of upregulated gene functions in those neurons and glia were related to synapse organization, neurotransmitter and dendrite development (Supplementary Fig. S6h). Generally, a common gene function enrichment could be observed in major cell lineages and specific cell types (Supplementary Fig. S6i). Global gene signatures and DEGs in different brain cell types shared comparable detection efficiency.

### Gene regulatory network in the normal mouse brain

**Fig. 3:**
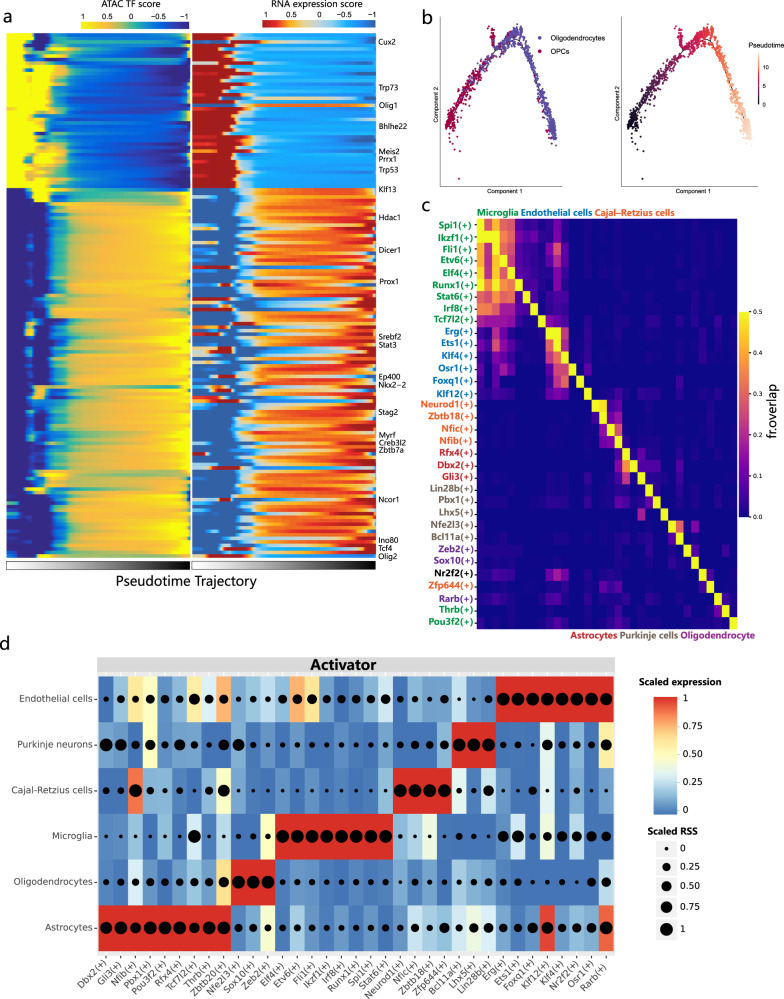
GRN analysis of normal mouse brain. **a** Heatmap of scaled expression scores and chromatin accessibility of representative TFs along the oligodendrocyte pseudotime trajectory. **b** Pseudotime analyses of the oligodendrocytes and OPCs. **c** Heatmap showing the overlap between target regions of cell type-specific regulons. The overlap is divided by the number of target regions of each regulon (row). The TF overlap is evaluated by calculating the Jaccard index. **d** Heatmap and dot plot showing the expression of the inferred activator regulons. The expression level is color-coded. The cell type specificity (RSS) of the regulons is coded by dot size.

We further investigated whether Microwell-seq3 datasets would allow for gene regulatory network constructions. We used Single-Cell gene Regulation network Inference using ChIP-seq and motif (SCRIP)^47^ to infer transcriptional regulators and trajectories by integrative analysis of our adult mouse brain datasets. To reconstruct the oligodendrocyte differentiation process, we first used the FNN package to transfer the pseudotime trajectory value of oligodendrocytes from the RNA-seq to the ATAC-seq data. We leveraged both the SCRIP ATAC-seq enrichment matrix and RNA-seq pseudobulk matrix to estimate the trajectory stage and common TFs. Oligodendrocytes were arranged along the pseudotime trajectory at the early and late stages (Fig. 3a). The RNA expression level of common regulators in oligodendrocytes was consistent with the TF activity score in ATAC-seq data. Oligodendrocyte differentiation-associated TFs, including *Olig1* and *Olig2*, were identified along the pseudotime trajectory. In the early stage, *Tp53* and *Tp73* are essential for cell cycle arrest and p53 family-dependent differentiation of oligodendrocyte precursor cells (OPCs)^48^. *Prrx1* induces stem cell quiescence in human and mouse OPCs^49^. In the late stage, coherent gene module networks of epigenetic genes drive the maturation of oligodendrocytes. Oligodendrocyte lineage determinants *Sox10* and *Myrf* recognize the regulatory regions of *Klf13* to promote the expression of myelin genes^50^. Joint maintaining of *Nkx2.2* and *Olig2* is targeted by *Sox10* during premyelinating of oligodendrocyte^51^. *Tcf4* is a preferred heterodimerization partner of *Olig2* in oligodendrocyte differentiation^52^. Other late stage-enriched TFs, such as *Prox1* and *Dicer1*, are required for oligodendrocyte differentiation in different manner^53,54^. Reconstruction of gene expression data also arranged OPCs and oligodendrocytes in another independent pseudotime trajectory (Fig. 3b; Supplementary Fig. S7a). In summary, chromatin remodeling marks the transition of oligodendrogenesis. The cell differentiation trajectory could be successfully reconstructed by chromatin accessibility regulator analysis integrated with gene expression data.

We then used single-cell regulatory network inference and clustering plus (SCENIC+ )^55^ to infer the gene regulatory network (GRN) in the whole mouse brain. SCENIC+ integrates chromatin accessibility and gene expression data to improve the accuracy of TF binding site predictions. We recovered cell type-specific enhancer regulons of astrocytes (*Dbx2*, *Rfx4*), endothelial cells (*Ets1*, *Osr1* and *Klf4*), microglia (*Runx1*, *Irf8*), oligodendrocytes (*Zeb2*, *Sox10*, *Nfe2l3*) and different neuron subtypes (*Neurod1*, *Lhx5*, *Bcl11a*, *Nfib* and *Nfic*) (Supplementary Fig. S7b). Synergistic binding patterns were not observed among the cell type-specific TFs (Fig. 3c). Scaled expression of TFs in the SCENIC+ results allowed the classification of activated TFs that have positive correlations with target region accessibility and target gene expression (Fig. 3d). In this case, the combination of multimodal data and suitable GRN tools accurately detected key regulators. These results could be used to further infer the cooperativity between TFs and enhancers.

### Identification of malignant cells in spontaneous lung tumors from aged mice

**Fig. 4:**
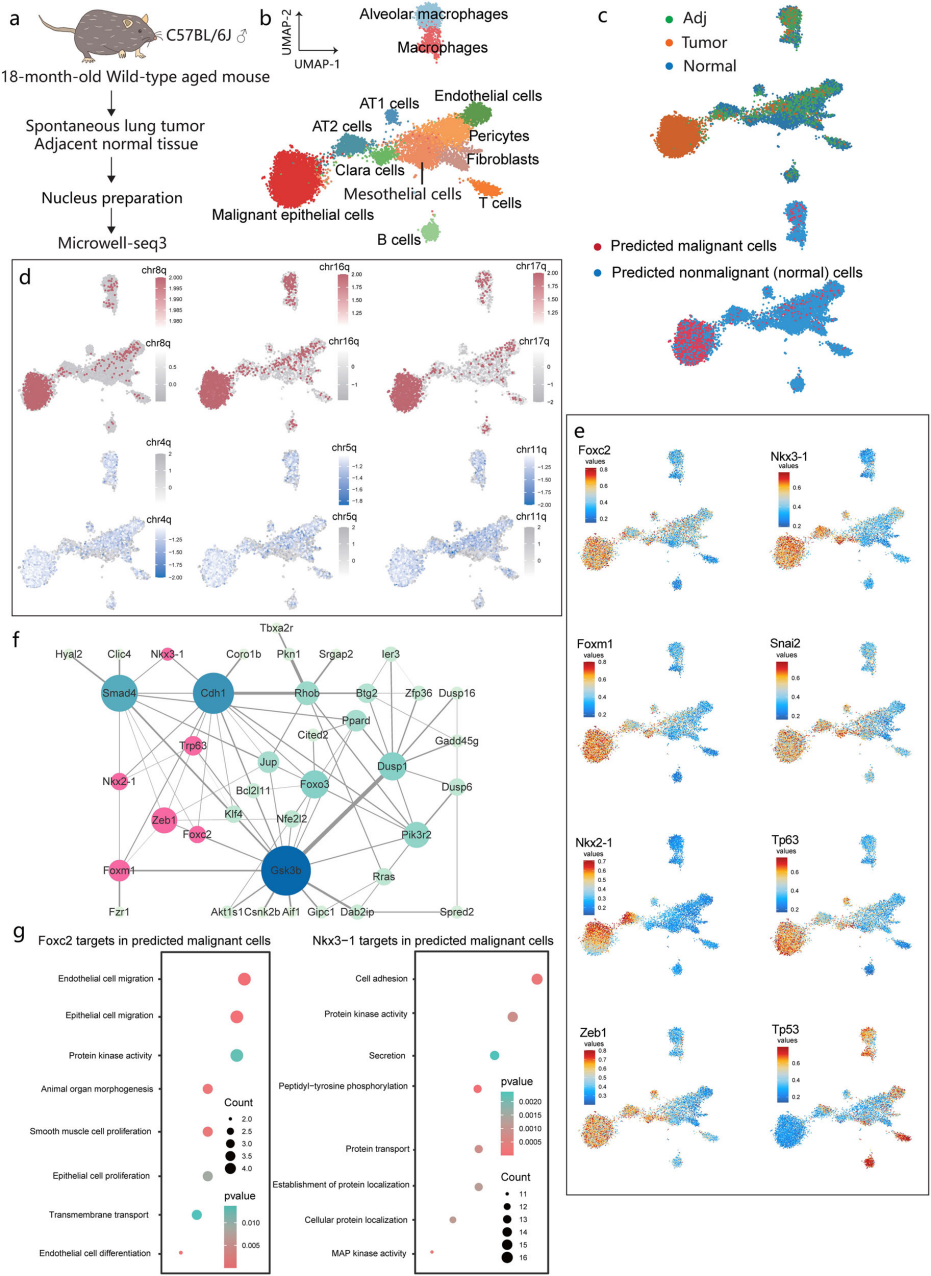
Analysis of spontaneous lung tumors in aged mice. **a** Workflow for analysis of spontaneous lung tumors in mouse using Microwell-seq3. **b** UMAP plot of cell types from paired normal lung tissue, spontaneous lung tumor tissue and tumor-adjacent tissue. **c** UMAP plot showing the distributions of tissue type (upper) and predicted malignant cells. **d** UMAP plot showing the gain and loss of specific chromosomes in tumor tissues. The gray bar represents the nonsignificant range. **e** UMAP plot of the enrichment scores for the most specific TFs in tumor tissues. **f** Network showing the correlations between the most specific TFs and their target genes. Selected TFs are labeled pink. The color depth of the target genes represents the node degree. The node size represents the number of the connected genes. **g** Gene ontology enrichment analysis of the target genes of *Foxc2* and *Nkx3.1* in the predicted malignant cells.

**Fig. 5:**
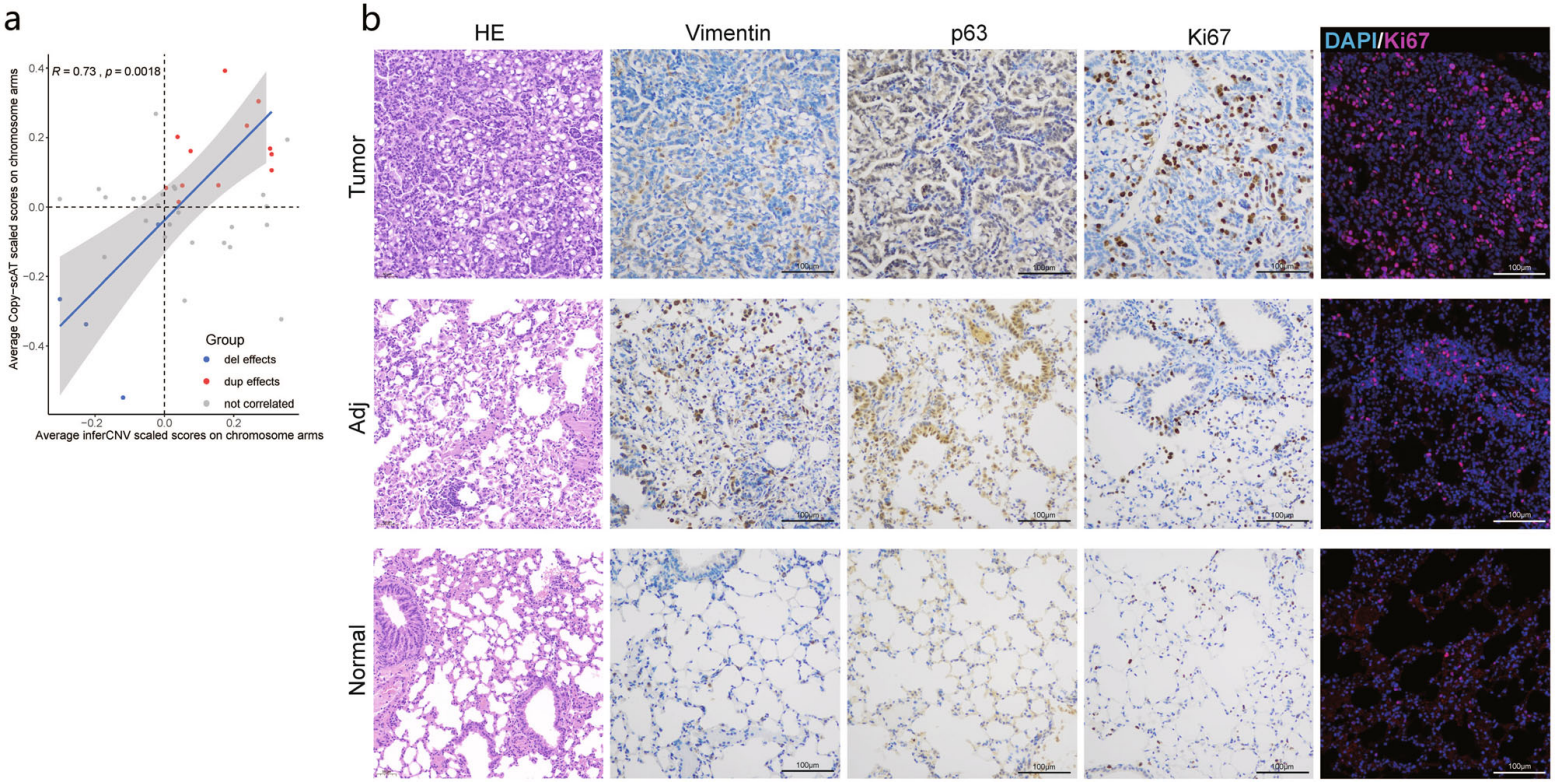
CNV correlation analysis and validation of the malignant signatures. **a** Correlation analysis of chromosome level CNV results between RNA-seq and ATAC-seq data. Only duplication effects (dup effect) and deletion effects (del effect) in overlapped the cytobands (chromosome region, see Materials and methods) were included for correlation analysis. Cytobands with inconsistent trend across different methods were labeled as “not correlated”. **b** Representative images of HE, IHC and IF staining results of tumor specific markers in tumor, tumor-adjacent tissue (Adj) and normal tissue sections. Scale bars, 100 μm.

Single-cell sequencing has been used to clarify the cancer cell heterogeneity in patients after treatment^56^. However, substantial variations in cell type-specific chromatin accessibility have not been fully linked to the intratumoral state of parenchymal cells^57^. To explore the scenarios in which our method could be applied to assist with clinical diagnosis, for example, tumor classification, we performed Microwell-seq3 to identify malignant cells in spontaneous lung tumor from aged mice (Fig. 4a). Age-related tumorigenesis in humans is partially reproduced in spontaneous tumors in wild-type aged mice^58^. The single-cell chromatin accessibility data of spontaneous lung tumor, tumor-adjacent tissue from an aged mouse were integrated with normal lung data. Classification of snATAC-seq clusters revealed a comprehensive set of cell types, including alveolar type I cells (AT1 cells), alveolar type II cells (AT2 cells), Clara cells (club cells), mesothelial cells, fibroblasts, endothelial cells and pericytes, B cells and T cells, macrophages and alveolar macrophages (Fig. 4b). Notably, we observed a cluster of tumor-specific epithelial cells with transitional identity between alveolar epithelial cells and mesenchymal cells (Fig. 4c). To identify malignant cells in tumor tissue, we estimated CNVs from snATAC-seq data using Copy-scAT^59^ and Alleloscope^60^. These two independent methods were used to infer potential global genome alterations in tumor tissues (Supplementary Fig. S8a, b, d). The predicted malignant cells were also enriched in tumor-specific cluster (Fig. 4c; Supplementary Fig. S8c). The ATAC-seq signals in tumor-specific clusters showed amplification of regions on chromosomes 8, 16 and 17. Malignant cells with loss of regions on chromosomes 4, 5 and 11 were enriched in tumor and tumor-adjacent tissues (Fig. 4d). We also evaluated CNVs in paired snRNA-seq data using inferCNV^61^. Consistently, we observed loss of regions on chromosomes 4, 5, 11 and amplification of regions on chromosomes 8, 10, 12, 16 and 18–19 (Supplementary Fig. S9a). At chromosome level, duplication and deletion patterns (see Materials and methods) in RNA-seq data also correlated well with ATAC-seq data (Fig. 5a) (Supplementary Table S5). The CNV score for each cell in tumor and tumor-adjacent tissues was classified into different levels to define malignant cells (Supplementary Fig. S9b). We observed cell state transitions (from normal to malignant) of epithelial cells based on the number of cells in the predicted state and CNV scores, among which myoepithelial cells and AT2 cells constituted a majority of malignant cells. Malignant cells in the myoepithelial cell and AT2 cluster also exhibited a significant high CNV score (Supplementary Fig. S9e).

We next identified activated TFs in predicted malignant cells. The tumor tissue-derived epithelial cell cluster exhibited high activity of the AT2 cell marker *Nkx2-1* (Fig. 4e). Compared with cells derived from normal tissue, cells derived from tumor and tumor-adjacent tissues exhibited high enrichment scores for *Foxc2*, *Nkx3-1*, *Foxm1*, *Tp63* and *Zeb1*, while *Tp53* activity was relatively enriched in normal tissue. Consistent CNV and gene expression patterns of those TFs were detected in gene expression data (Supplementary Fig. S9c, d). *Foxc2*, which is located on chromosome 8, regulates the epithelial-mesenchymal transition process during metastasis^62^. The transient expression pattern of *Nkx3.1* in the mesenchyme cells regulates stemness of transitional malignant epithelial cells during metastasis^63^. SCRIP analysis was utilized to construct a potential target gene network of major regulators in malignant cells (Fig. 4f). One of those hub genes was the common tumor suppressor *Cdh1*, which is correlated with the self-renewal of lung cancer cells and promotes mesenchymal-to-epithelial transition during the colonization phase^64^. Gene ontology enrichment analysis of the target genes of the key regulatory hubs such as *Foxc2* and *Nkx3.1* showed enrichment of cell adhesion, epithelial and endothelial cell migration, protein kinase activity and cell migration (Fig. 4g; Supplementary Table S4). Thus, malignant epithelial cells in spontaneous lung tumors demonstrated stemness characteristics of AT2 cells and a transitional state between mesenchymal cells and alveolar epithelial cells. Gene set variation analysis (GSVA) of tumor tissue-derived myoepithelial cells also showed transitional function enrichment state of tumor associated signaling pathways (Supplementary Fig. S9f), indicating the distinct transitional process in the malignant cells. Furthermore, hematoxylin and eosin (HE) staining showed malignant morphology of cells in tumor samples. Immunohistochemistry (IHC) and immunofluorescence (IF) stainings also indicated upregulation of lung tumor-associated markers including *Ki67* and *p63* in the tumor tissues compared to the tumor-adjacent and normal tissues (Fig. 5b). These results suggested the potential application of Microwell-seq3 to elucidate the characteristics of malignant cells in tumor samples.

## Discussion

Commercialized single-cell multi-omics platforms accelerated our study of different molecular and cellular layers in the biological systems^65^. The limitations of expensive microfluidic devices cumber their application in different labs. Other commercialized microfluidics-free methods (Parse Bioscience and Fluent Bioscience, PIP-seq^66^) provided alternatives with no requirement of specialized devices. We previously reported another instrument-free agarose microwell plate methods for single-cell sequencing with high cost efficiency and cell type compatibility^22,36^. Here, we report Microwell-seq3 for both snRNA-seq or snATAC-seq. In this version, we adopted a penetrable designed chip-in-a-tube microwell to greatly simplify the loading step (manual manipulation time of each chip < 1 min) (Supplementary Methods). Combining preindexing and bead barcoding strategies, our method provides large-scale barcode combinations to tag different samples in one experiment.

The compelling potential advantages of this method also include the ability for multiple rounds of reagent loading. The penetrable well traps the reagents and cells, as surface liquid can be evaporated in the incubator during the reaction (or using a desktop vacuum pump to accelerate the process). Thus, compared with the sealed microfluidics system, Microwell-seq3 shows flexible ability and is tailored to other omics study designs that require multiple reaction steps after single-cell isolation. To take advantage of this exclusive feature, one can perform extended antibody-labeling-based applications such as ChIP-seq, CUT&Tag^67^ and CITE-seq^13^ in a reagent-saving manner. Non-commercial single-cell sequencing methods based on combinatorial indexing have provided instrument-free strategies for RNA-seq (sci-RNA-seq^25,26^, SPLiT-seq^24^), ATAC-seq (sci-ATAC-seq^68^, s3-ATAC^42^) and multi-omics (sci-CAR^69^, Paired-seq^70^, SNARE-seq2^71^, CoTECH^72^). Nevertheless, the stability and validity of the time-consuming combinatorial indexing methods depend on experimental skills (or using an automated benchtop pipettor). Microwell-seq3 only adopts one round of preindexing to improve throughput and label different samples. This method greatly simplifies the loading step compared with microfluidic system or other microwell platforms. Thus, Microwell-seq3 is a low-cost, time-saving and extendable platform for single-cell omics profiling.

High-sensitivity single-cell chromatin accessibility data are suitable for recently developed GRN construction tools including SCRIP^47^ and SCENIC+ ^55^. Furthermore, we revealed the ability for the identification of malignant cells in spontaneous lung tumors using Microwell-seq3 multi-omics data. GRN construction based on single-cell chromatin accessibility data demonstrated better regulon enrichment performance than gene expression data. One limitation of the current Microwell-seq3 RNA-seq protocol is the low proportion of immune cells due to the combination of single nucleus preparation and fixation strategy^73^. The potential solution for immune cells-enriched tissues such as spleen and bone marrow, and peripheral blood mononuclear cells is to use the reversible fixation of single cells rather than nuclei in the preindexing step^74^. Thus, the simple and rapid workflow of our method has the potential to be used for clinical diagnosis with small frozen or fresh samples. No special equipment is required for any part of the process, and the process can be performed in different laboratories or institutions in an efficient manner.

## Materials and methods

### Cell lines and culture

Mouse NIH3T3 (RRID: CVCL_0594) and human HEK293T (RRID: CVCL_0063) cells were cultured in Dulbecco’s modified Eagle’s medium (DMEM, Gibco) supplemented with 10% fetal bovine serum (FBS, Thermo Fisher) and 1% penicillin-streptomycin (Thermo Fisher). Cells were passaged every 2 days and cultured in 6-well culture plates. Cells were harvested by trypsinization (0.25%, Thermo Fisher) treatment followed by pelleting by centrifugation at 300× *g* for 5 min. After aspiration of the supernatant, the pellet was washed twice using cold PBS. Finally, the cells were resuspended in cold 1× PBS and counted for further nuclear extraction.

### Mice

Wild-type C57BL/6 J mice (8-week-old as young and 18-month-old as aged) were ordered from Beijing Vital River Laboratory Animal Technology Co., Ltd. All mice were housed at the Zhejiang University Laboratory Animal Center in a specific pathogen-free facility in individual ventilated cages. The room had controlled temperature (20–22 °C), humidity (30%–70%) and light (12 h light-dark cycle) conditions. Mice were provided ad libitum access to a regular rodent chow diet. All tissues were freshly collected before nuclear extraction. Tumors and tumor-adjacent tissue were harvested from male mice. All the animal experiments performed in this study were approved by the animal ethics committee of the First Affiliated Hospital, Zhejiang University School of Medicine (approval number: 2023 No.72). All experiments conformed to the relevant regulatory standards of Zhejiang University Laboratory Animal Center.

### Nucleus preparation

For cell lines, single cells were resuspended in cold 1× PBS and pelleted by centrifugation at 300× *g* for 5 min. The cell pellet was lysed in 1 mL of ice-cold lysis buffer (for RNA assays: 10 mM Tris-HCl (pH 7.4), 10 mM NaCl, 3 mM MgCl_2_, 0.1% Tween 20, 1% Murine RNase Inhibitor (Vazyme Biotech), 0.1% IGEPAL CA-630, 1 mM DTT; for ATAC-seq: 10 mM Tris-HCl (pH 8.0), 10 mM NaCl, 3 mM MgCl_2_, 0.1% Tween 20, 0.1% IGEPAL CA-630, 0.01% digitonin (Promega), 1× protease inhibitor (Sangon Biotech)) and incubated on ice for 2 min. Then, 5 mL of ice-cold RSBT buffer (10 mM Tris-HCl (pH 7.4), 10 mM NaCl, 3 mM MgCl_2_, 0.1% Tween 20) was added to terminate the lysis reaction. The medium was then filtered using a 40-μm strainer. Nuclei were centrifuged at 500× *g* for 5 min at 4 °C and washed with 1 mL of cold RSBT buffer. For RNA assays, nuclei were resuspended in 100 µL of RSBT buffer, and 10 mL of ice-cold 4% paraformaldehyde was then added carefully. Nuclei were fixed at 4 °C for 15 min, and 1.5 mL of 2.5 M glycine (Sangon Biotech) was added to quench the fixative. Fixed nuclei were washed twice with 1 mL of RSBT buffer (by centrifugation at 500× *g* for 5 min at 4 °C). To freeze nuclei, fresh or fixed nuclei were resuspended in cold freezing buffer (50 mM Tris-HCl (pH 8.0), 25% glycerin, 5 mM magnesium acetate, 0.1 mM EDTA) and stored at –80 °C. Fresh mouse tissues were cut and ground in liquid nitrogen using a stainless-steel blender. The cold tissue powders were rapidly transferred into a 1.5-mL centrifuge tube. The nucleus isolation step was the same for cells from cell lines and tissues. Nuclei isolated from tissues were centrifuged at 800× *g* for 5 min in all centrifugation steps. For counting of nuclei, 9 µL of nuclear suspension was mixed with 1 µL of 10× Ultra GelRed (Vazyme Biotech, cat# GR501) and loaded into the cell counting chamber.

### Microwell-seq3-RNA: in situ reverse transcription

The protocols for reverse transcription of RNA from fixed nuclei were similar to those for preindexing of the cytoplasmic transcriptome^75^. Nuclei were resuspended in reverse transcription buffer mix (each well: 0.5 µL 10 mM dNTPs, 0.5 µL 10% Triton X-100, 1.5 µL PEG8000 (Sigma-Aldrich), 3.6 µL of fixed nuclei (10,000 nuclei) in RSB buffer without Tween-20) and partitioned into one or more (up to 4) 96-well plates. Then, a 1 µL volume containing a 10 µM concentration of 384 barcoded random primers (Supplementary Table S1) was added to each well. The plates were incubated at 55 °C for 5 min and immediately placed on ice. Then, 3 µL of reverse transcription enzyme mix (2 µL of 5× RT buffer (31 mM Tris-HCl (pH 8.0), 37.5 mM NaCl, 3.1 mM MgCl_2_, 10 mM DTT), 0.5 µL of Murine RNase Inhibitor, 0.5 µL of Maxima H Minus RTase (Thermo Fisher)) was added into each well. The in situ reverse transcription reaction was performed as follows: ten cycles of 8 °C for 12 s, 15 °C for 30 s, 20 °C for 45 s, 25 °C for 1 min, 30 °C for 1 min, and 42 °C for 2 min; followed by a final extension step at 42 °C for 30 min.

### Microwell-seq3-RNA: exonuclease I treatment

After reverse transcription, the 96-well plates were placed on ice for 2 min to stop the reaction. Then, 3 µL of Exonuclease I mix (1.3 µL of 10× Exonuclease I Reaction Buffer (NEB), 1.5 µL of nuclease-free water, 0.2 µL of Exonuclease I (NEB)) was added into each well. The plates were slowly rotated at 37 °C for 30 min in an incubator.

### Microwell-seq3-RNA: poly(A) tailing

After Exonuclease I treatment, nuclei were pooled and washed twice with RSBT. Then, nuclei were resuspended in poly(A) tailing mix (20 µL of nuclei in RSB buffer, 56 µL of nuclease-free water, 2 µL of Murine RNase Inhibitor, 10 µL of 10× terminal transferase reaction buffer (NEB), 10 µL of 10×;CoCl_2_ (NEB), 5 µL of 100 mM dATP (Sangon Biotech), 1 µL of terminal transferase (NEB)). The tube was slowly rotated at 37 °C for 15 min in an incubator. After the dA-tailing reaction, the nuclei were washed with 1 mL of 3× SSC-T (3× SSC, 0.05% Tween 20) and twice with 1 mL of RSB buffer. The nuclei were resuspended in RSB buffer before loading on the chips.

### Microwell-seq3-ATAC: Tn5 transposase complex assembly

For Tn5 transposase complex assembly, Tn5_primer_A, 384 barcoded Tn5_P5primer_B and Tn5_P7primer_C with MGI adapter (Supplementary Table S1) were dissolved in TE buffer (10 mM Tris-HCl, 0.1 mM EDTA; pH8.0) to a final concentration of 100 μM. Next, Tn5_primer_A, barcoded Tn5_P5primer_B and Tn5_P7primer_C were mixed at ratio of 2:1:1 in four 96-well plates. The plates were placed in thermocyclers and oligos were annealed as follows: 95 °C for 2 min, followed by slow cooling to 20 °C with a temperature ramp of –0.1 °C/s. The annealed mixture was diluted to a final concentration of 1.4 μM in each well. Then, 3.75 µL of Tn5 transposase mix (10 µL of Tn5 (Vazyme Biotech, cat# S111), 33 µL of coupling buffer (Vazyme Biotech, cat# S111), 332 µL of dilution buffer (Vazyme Biotech, cat# S111)) and a 1.25 µL volume containing 1.4 μM concentration of the annealed mixture were added to four 96-well plates. The plates were incubated at 25 °C for 1 h. The final Tn5 transposase-containing plates were stored at –20 °C.

### Microwell-seq3-ATAC: tagmentation

Nuclei were washed with RSBT buffer and counted. Then, 2× TD buffer (20 mM Tris-HCl (pH7.4), 10 mM MgCl_2_, 20% dimethylformamide (Sigma-Aldrich)) was prepared and stored at 4 °C before tagmentation. Then, nuclei were resuspended in tagmentation mix (25 µL for each well: 12.5 µL of 2× TD buffer, 8 µL of 1× PBS, 0.25 µL of 10% Tween 20, 0.25 µL of 1% digitonin, 2 µL of the assembled Tn5, 2 µL of nuclease-free water) and partitioned into four 96-well plates (10,000 nuclei in each well). Tagmentation was performed at 55 °C for 30 min. The plates were then placed on ice for 5 min to stop the reaction. Nuclei were pooled and washed twice with RSB buffer before loading on the chips.

### Microwell-seq3: microwell chips and synthesis of barcoded beads

Microwell chips were obtained from Clarity™ Digital PCR system (JN Medsys, cat# 12007, agent by Neoline Technology Co., Ltd.), and custom microwell chips were fabricated based on ZZ-Bio Digital PCR System chips (ZHENZHUN Biotechnology Co., Ltd.). Every chip in the 0.2 mL tube contained 10,000 partitions. The total reaction volume in one chip was ~15 µL. The diagonal length of a hexagonal partition is 60 µm. The 20 µm carboxyl modified magnetic beads (50 mg/mL) were obtained from SuZhou KBsphere Co., Ltd. (cat# MagCOOH-20190911). The three-step protocol for the synthesis of the oligo barcoded magnetic beads was the same as that used in Microwell-seq 1.0^22^. The 96 beads_primer_A oligos contained the first part of the cell barcode and the uracil base modification (Supplementary Table S1). The 96 beads_primer_B oligos contained the second part of the cell barcode. The 96 RNA_beads_primer_C oligos contained the third part of the cell barcode, the UMI and a ploy(T) tail to capture poly(A) tailed transcripts. The 96 ATAC_beads_primer_C oligos contained the third part of the cell barcode and a tagmentation hybridization linker to capture DNA fragments.

### Microwell-seq3-RNA: chip loading and library preamplification

Approximately 20,000 nuclei and 20,000 barcoded beads were loaded onto one chip. The beads were washed twice with RSB buffer before use. The nuclei and beads were resuspended in loading mix, and 11 µL aliquots of loading mix with nuclei and beads (6 µL of nuclease-free water, 5 µL of KAPA HiFi HotStart ReadyMix (Roche)) were generated. The chip was taken out from the tube using a flat tip tweezers. The loading mix with nuclei and beads was pipetted a few times and quickly added to the chip surface using a flat end pipette tip. After all the liquid was inhaled into the chip (5–10 s), another 5 µL of loading enzyme mix (0.8 µL of Bsu DNA Polymerase, Large Fragment (NEB); 0.6 µL of RNase H (NEB); 0.6 µL of USER enzyme (NEB); 3 µL of KAPA HiFi HotStart ReadyMix) was added into the chip. Next, 150 µL of sealing oil was carefully added along the tube wall to seal the chip. The bead oligo cleavage and linear amplification reactions were performed in the chip under the following conditions: 37 °C for 1 h, 72 °C for 30 min, and 98 °C for 10 min; ten cycles of 98 °C for 3 min, 55 °C for 3 min, and 72 °C for 4 min; followed by a final incubation step at 72 °C for 30 min.

### Microwell-seq3-ATAC: chip loading and library preamplification

The loading procedures for ATAC-seq were the same as those for RNA-seq. Loading mix with nuclei and beads: 2 µL of 5× KAPA HiFi Fidelity Buffer (Roche, cat# KB2500), 1 µL of 10 mM KAPA dNTP mix, 7 µL of 50 mM EDTA, 3.7 µL of RSB buffer with nuclei and beads, 0.3 µL of 100 µM ATAC_SpikeIn_oligos (Supplementary Table S1). After all the liquid was inhaled into the chips (5–10 s), the chips were put back into the tubes, and the tube lids were covered. The tubes were placed into a 50 °C incubator for 30 min to release the DNA fragments from the nuclei. Then, another 6.6 µL of loading enzyme mix (2 µL of 5× KAPA HiFi Fidelity Buffer, 1 µL of nuclease-free water, 1 µL of 1U/µL KAPA HiFi HotStart DNA Polymerase (Roche, cat# KE2502), 2 µL of 400 mM MgCl_2_, 0.6 µL of USER enzyme) was added into the chip. The procedures for oil sealing and the subsequent linear amplification reaction in the chip were the same as those used in the RNA-seq protocol.

### Microwell-seq3: final library amplification

For both RNA-seq and ATAC-seq, the sealing oil was gently removed after the preamplification step. Then, 80 µL of nuclease-free water with 6× DNA loading dye (Thermo Fisher) was directly added into the chip. The tubes containing the chips were put into a high-speed bench top centrifuge and centrifuged for 5 min to collect all the liquid in the chips. The liquid with loading dye was separated and transferred to another new PCR tube strip. The tubes were placed on a magnetic stand to remove all the beads. For each chip, the preamplified library was purified using 1.5× VANTS DNA cleaning beads (Vazyme Biotech). Then, 18 µL of the purified preamplified library was mixed with 1 µL of MGI_P5_primer (Supplementary Table S1), 1 µL of MGI_P7_primer and 20 µL of KAPA HiFi HotStart ReadyMix. PCR was performed with the following thermal cycling program: 72 °C for 5 min; 98 °C for 3 min; twelve cycles of 98 °C for 20 s, 60 °C for 30 s, and 72 °C for 1 min; 72 °C for 5 min; and holding at 10 °C. The final RNA library was purified using 1.2× VAHTS DNA cleaning beads. The final ATAC-seq library was purified using two rounds of size selection: 0.55× VAHTS DNA cleaning beads was used for the first round, and the supernatant was collected; 1.0× VAHTS DNA cleaning beads was used for the second round.

### MGI library preparation and sequencing

The concentration of the final library was determined with a Qubit 3.0 fluorometer (Invitrogen). The purified linear DNA library was circularized to generate a single-stranded DNA (ssDNA) library using a VAHTS Circularization Kit for MGI (Vazyme Biotech). The ssDNA library was amplified using a DNBSEQ DNB preparation kit (MGI). Amplified DNA nanoballs were sequenced on the MGI DNBSEQ-T7 platform using paired-end 150 bp mode. Custom TM (Tn5 modified) sequencing primers were adopted (Supplementary Table S1).

### Preprocessing of Microwell-seq3 data

Raw reads were preprocessed with custom scripts. The 6-nt UMI and 18-nt cell-specific barcodes in each Read1 and the 10-nt preindex barcodes in each Read2 were extracted using umi-tools (version 1.1.2)^76^. In RNA-seq, we used STAR (version 2.5.2a)^77^ with default parameters to align reads from HEK293T cells and NIH3T3 cells to the designed hg38-mm39 reference genome, and reads from mice were aligned to the GRCm38-mm10 reference genome. Paired GTF annotation files were used to tag aligned reads. A list of cell barcode oligo sequences were used to correct the cellular barcodes extracted from each Read1. Finally, digital expression matrices were obtained using Drop-seq tools (version 2.5.1)^19^. In the species-mixing experiments, after low-quality cells with < 500 transcripts were discarded, cells with a percentage of reads (over 80% of the UMIs) uniquely mapped to the genome of either species were regarded as species specific, while the remaining cells were labeled collisions. In the quality control step for data from tumor samples, tumor-derived cells with < 300 genes were discarded, and cells from tumor-adjacent tissues were retained. Doublets were identified by the R package scDblFinder with the filter ratio set to 0.1.

For snATAC-seq, reads from mice were converted to fastq format and aligned to the mm10 reference genome using bwa (version 0.7.15)^78^. Then, the 18-nt cell-specific barcodes and 10-nt preindexes were directly extracted from each Read1. One mismatched barcode in the alignment was corrected by the same list of barcode oligo sequences used in the RNA processing procedure. Fragments were obtained from aligned BAM files by SnapTools (version 1.4.8)^79^ and custom Python scripts. To save time and space, we retained only the 20,000 barcodes (10, 000 for primary tumor samples) with the highest number of unique fragments for downstream analysis. In the species-mixing experiments, HEK293T cells and NIH3T3 cells were mapped to the hg38-mm39 reference genome using bwa. Cells with < 3000 reads were defined as low-quality cells, and cells with >80% of reads uniquely mapped to either genome were regarded as species-specific cells, while the remaining cells were labeled collisions. For each fragment file, a cell-bin matrix with a trunk size of 5000 was generated using ArchR (version 1.0.2)^80^ for dimension reduction and UMAP visualization. For tumor samples, cells with < 800 unique fragments and a TSS enrichment score < 4 along with the potential doublets were removed.

### Identification of “cell-containing wells with beads” in a chip

We adopted a published method to determine the “Cell-containing wells with beads” marked by single or multiple beads and at least one nucleus^11^. In the published work, this method was aim to identify the overloaded bead which belongs the same droplet. Here in our study, the first step was also to exclude the empty wells without a bead and arrange the beads into different “Cell-containing wells with beads”. Bead barcodes sharing a noticeable overlap of random oligonucleotides would be barcodes from two beads contained in the same well. In snRNA-seq data, we directly used the 17-base (10-base UMIs and 7-base random oligonucleotide) sequence in RNA-seq library as marked random oligonucleotides. In snATAC-seq data, we introduced the spike-in library containing a 14-base random oligonucleotide (Supplementary Table S1). We used these random oligonucleotides to compute the Jaccard index over the reads that shared the same feature sequences for the top 20,000 barcodes (BC#1 and BC#2) ranked by reads-UMIs (RNA-seq) and reads-fragments (ATAC-seq) in matrix files. Then a knee-calling algorithm (bap2) was performed to establish a threshold to determine bead pairs that were likely originated from the same well based on the Jaccard index. We further removed wells with >6 bead barcodes, reasoning that these represented technical confounders. Filtered bead pairs were looped over to assign the same bead barcode (BC#2). After beads merging, the second step was to restore the reads in each single nucleus because there may also have multiple nuclei in the same well. Reads from the bead barcodes were simply assigned to different RT barcodes or Tn5 barcodes (BC#1). These merged barcodes (processed BC#1 with assigned BC#2) were the final single-cell barcodes. Processed bam files with merged barcodes were used to generate digital expression matrix files (RNA-seq) or fragment files (ATAC-seq) for downstream analysis.

### Microwell-seq3 RNA data analysis

The gene expression UMI matrix was log-normalized and scaled with a scale factor of 10,000 following the Seurat package’s standard workflow. Principal component analysis was used for dimensional reduction of the data, and the first 50 dimensions were retained to identify the cell clusters by the Louvain clustering method. UMAP was used to visualize the clustering results. Markers from each cluster were identified by the ‘FindAllMarkers’ function with default parameters. We annotated each cluster based on canonical marker genes in other single-cell studies and datasets^22,81,82^. Similar processes were performed with adapted parameters for analysis of each tissue.

### Microwell-seq3 ATAC data analysis

Cell-bin matrices with a trunk size of 5000 from different chips were merged and analyzed uniformly with the standard workflow of ArchR. For data from wild-type mice, cells with a TSS enrichment score < 7 or < 1000 unique fragments were removed from the analysis, along with potential doublets. In the quality control step for data from primary mouse tumor, doublets and cells with < 1000 unique fragments were discarded. We applied an iterative LSI dimensionality reduction technique to obtain a low dimensional representation of single-cell ATAC datasets. Clustering was then performed using the ‘addClusters’ function, and a UMAP plot was generated using ‘addUMAP’ (minDist = 0.4). Markers from each cluster were identified by a wrapped function using gene scores generated by ArchR with threshold criteria of FDR < 0.1 and |log_2_FC| > 1. In addition to annotating cell types by identified marker genes in previous studies, we performed label transfer with our ATAC-seq data and RNA-seq data from Microwell-seq3 RNA-seq and the Mouse Cell Atlas to ensure the accuracy of our annotations. In the species-mixing experiments, fragments from HEK293T cells and NIH3T3 cells were separated by chromosome names and peak calling was performed on the aggregated reads using MACS2 (version 2.2.7.1)^83^. Peaks were annotated by the ChIPseeker package (version 1.26.2)^84^ with a TSS region set between TSS downstream 3000 and upstream 3000.

### Benchmarking, gene body coverage and gene biotype analysis

We used Smart-seq3 (E-MTAB-8735), VASA-plate (GSE176588), FLASH-seq (Sequence Read Archive, PRJNA816486), Smart-seq-total (GSE151334), 10X Genomics (10x Genomics official website), Microwell-seq2 (GSE175413) and Drop-seq (GSE63269) data for benchmarking. All the raw data were mapped to the hg38-mm39 genome using STAR (version 2.5.2) with default parameters after trimming of homopolymers and the extraction of beads barcodes and UMIs. The generated single-cell aligned bam files were merged using SAMtools (version 1.15)^85^. For the gene detection assay, only cells that had been sequenced to the highest numbers of reads were retained (15,000 reads for the saturation curve). Down-sampling was carried out using the aligned bam files. Only reads that mapped to coding and intronic regions were counted. The gene body coverage across the technologies was quantified by the geneBody_coverage.py module in the RSeQC package (version 4.0.0)^86^. Reads were trimmed to 5000 per cell and 500,000 per sample. To acquire the type of transcript, we used TagReadWithGeneFunction implemented in Drop-seq tools to annotate reads to mapped genes according to the corresponding GTF file. Reads mapped to multiple genes simultaneously were defined as ‘Multi’.

### Comparison between Microwell-seq3 RNA-seq and other 3′ end scRNA-seq data

To illustrate the properties of total RNA-seq, we quantified similarities and differences between RNA-seq data in Microwell-seq3, VASA-seq and 10X Genomics. Genes identified as expressed by all technologies were retained, and RNA counts were uniformly normalized to TPM. The correlation between Microwell-seq3 data and data obtained by other technologies was assessed by the Pearson correlation coefficient. We further compared the gene expression pattern in mouse brain between 10X Genomics and Microwell-seq3. We obtained external 10X Genomics scRNA-seq data of mouse whole brain (GSM6617915), 10X Genomics snRNA-seq of mouse cortex (GSE140511), 10X Genomics snRNA-seq of mouse cerebellum (GSE165371). 10X Genomics snRNA-seq data of mouse heart, kidney and liver are available on the official website. As for the comparison with 10X Genomics whole brain scRNA-seq data, only genes expressed in both technologies were used. The counts of genes were normalized to TPM. The DEGs were defined by a two-sided Student’s *t*-test with the threshold of |logFC| > 2 and *p*-value < 0.01. As for the comparison with 10X Genomics snRNA-seq data, only cells that had been sequenced to at least 1000 reads in coding and intronic regions were used to down-sampling. R package dscBlast (version 1.0.3) was then employed to assign cell types to the clusters identified in the integrated datasets of 10X Genomics mouse cortex and cerebellum. Genes with < 5 counts across all the cells were removed. Finally, the differential expression assay was carried out in 4 common cell types (interneurons, Purkinje neurons, astrocytes and oligodendrocytes). Wilcoxin test was used on cell counts and the DEGs were defined following the same threshold (|logFC| > 2 and *P*-value < 0.01). The enrichment of DEGs compared to those identified by 10X Genomics sequencing (|log_2_FC| > 2 and *p*-value < 0.01) in specific gene ontology terms was determined using clusterProfiler (version 3.18.1)^87^. We annotated gene biotypes to the mm10 genome Ensembl GTF (version 88).

### TF network construction and pseudotime trajectory analysis

We used SCRIP^47^ for TF enrichment network construction and pseudotime trajectory analysis. For analysis of brain and other tissues, cell clusters without distinct markers (set as ‘Unannotated’ in snATAC-seq data) were discarded, and the remaining cell-bin matrix was extracted from an ArchRProject and preprocessed by custom scripts. We followed SCRIP’s standard workflow and employed the ‘enrich’ function to compute the TF activities of each cell based on the overlap of its accessible regions with reference ChIP-seq data. The significance of TF enrichment was determined by a BH-adjusted *p*-value of < 0.01 and log_2_FC of >2. We manually set an upper threshold of minus log_2_ BH-adjusted *p*-value = 300 if the *p*-value itself was computed as zero for a better visualization. We adapted a previously described method in Trevino et al. ^88^. To obtain a paired pseudotime trajectory. For oligodendrocytes in our RNA data, we reclustered these cells and used Monocle2^89^ to construct a pseudotime trajectory from precursors to mature glial cells. Then, gene scores produced by ArchR was log-normalized and scaled as surrogates for gene expression in the cells profiled by snATAC-seq. To integrate the two independent omics datasets, the union of 2000 most variable genes in each modality was used as input to the FindTransferAnchors’ function in Seurat with reduction method ‘cca’ and parameter ‘k.anchor = 30’. We employed the FNN algorithm to match cells profiled in snATAC-seq by the gene scores produced by ArchR with their nearest 50 RNA-seq neighbors in the common CCA space, and each ATAC-seq cell-mapped pseudotime value was defined as the average of those of its RNA-seq neighbors. Based on the mapping relationship determined by FNN, corresponding RNA-seq pseudobulk transcriptomes were also constructed to reduce sparsity and for computational convenience. The dynamics of aggregate gene expression and TF activities computed by SCRIP as described above were visualized side-by-side along the trajectory.

### Gene regulatory network construction

To further investigate TF–gene interaction patterns, we followed the SCENIC+ ^55^ (version 0.1) workflow to identify possible activators and repressors enriched in neurons, glial cells and nonneuronal cell types. First, we used a cell-bin matrix constructed from our ATAC-seq data as input and preprocessed it with custom scripts. The pycisTopic module (version 1.0.1) in SCENIC+ with default parameters was used to discriminate different chromatin accessibility states in each cell, and 15 cis-regulatory topics determined by the CGS model were assigned in this step. The pycisTarget module (version 1.0.1) in SCENIC+ was then used to mine underlying motifs based on the cis-regulatory topics found by pycisTopic and differentially accessible regions between each cell type, which could also be obtained through the standard workflow of pycisTopic. We used the mm10 motif database as a reference. Finally, SCENIC+ was employed to pair ATAC-seq data with RNA-seq data to jointly predict regulatory TFs based on the concordance among the accessible TF binding site, TF expression, and target gene expression. TFs with a TF-region and TF-gene correlation of > 0.5 were retained for further analysis and visualization.

### CNV analysis

#### Construction of pseudobulks for snRNA-seq and snATAC-seq data

To reduce the effects of sparsity and heterogeneity at the single-cell level, a similarity-based method was carried out to group the cells from different modalities and cell types into pseudobulks. First, cells were grouped by the sample source (from tumor, tumor-adjacent tissue, or wild-type normal tissue) and cell type. Groups with fewer cells (fewer than the number of pseudobulks) were discarded. The number of pseudobulks for each group was assigned manually. Pseudobulks were constructed by clustering the Euclidean distances between the counts of cells (counts of genes for RNA-seq; counts at each bin across the genome for ATAC-seq). Counts of each pseudobulk were the cumulative sum of cells and were normalized to 10e6.

#### Construction of normal references for snRNA-seq

R package inferCNV (version 1.14.0) and iterative hierarchical clustering were employed to infer normal and malignant cells in snRNA-seq data. We performed this analysis on the hypothesis that there were normal or malignant cells in tumor and tumor-adjacent tissues in reality. Initial CNVs for each region were estimated by inferCNV R package with tumor-adjacent tissues as reference. We adopted a published method to calculate the CNV score of each cell^90^. For each sample, gene expression level of pseudobulks was renormalized. Gene expression values were scaled to a range from –1 to 1. We calculated the CNV score of each pseudobulk as the quadratic sum of the CNV region. The mean CNV score of each tissue, denoting as “normal expectation” (defined by the tumor-adjacent tissue) and “malignant expectation” (defined by the tumor), was calculated as the mean value of all corresponding pseudobulks. Those results were corrected by removing pseudobulks with outliers (CNV scores ≥ malignant expectation in the tumor-adjacent tissue or ≤ normal expectation in the tumor). Hierarchical clustering was then employed to group pseudobulks into k clusters (*k* = 50). The mean CNV score per cluster was compared as follows:

1. mean CNV score ≤ normal expectation, defined as “normal”;

2. mean CNV score ≥ malignant expectation, defined as “malignant”;

3. mean CNV scores > normal expectation and < malignant expectation, defined as “intermediate”;

For all the pseudobulks which were defined as “intermediate” temporarily, a new round of clustering was performed to group these pseudobulks into k-clusters (*k* = 50). The CNV score of each cluster was recalculated. The clusters were further assigned to “normal” or “malignant” group. This iterative step would stop until no more “normal” or “malignant” clusters were generated, or reached the preset maximum recursion number. Finally, inferCNV was performed to calculate the CNV regions in the “normal” clusters with default setting. The prediction of cells in UMAP embedding was determined by the state of the pseudobulks.

#### Discern copy number alterations in snATAC-seq data

We employed the R package Copy-scAT^59^ (version 0.3.0) to discern large-scale copy number alterations and distinguish normal cells from malignant cells in snATAC-seq data. In order to make this method available for the mm10 genome, we downloaded the Cytoband and cpgIsland files from the UCSC Genome Browser. The whole genome was partitioned into windows of 1 million base pairs in length to summarize the total coverage from the pseudobulks fragment files. We incorporated normal mouse lung data to help distinguish the normal cells. A modified nonnegative matrix factorization algorithm and hierarchical clustering method implemented in the package (‘identifyNonNeoplastic’ function) were used to cluster the pseudobulks into k-clusters (*k* = 4) based on the shared CNV patterns in different chromosome cytobands (chromosome regions). We manually denoted all pseudobulks of wild-type mouse-derived cells as normal and labeled clusters with a ratio of wild-type pseudobulks above 80% as the normal cluster. Copy number alterations were then inferred in each pseudobulk using ‘identifyCNVCluster’ function with parameters minMix setting to 0.001, propDummy setting to 0.4, and median QuantileCutoff setting to –1. Likely unaltered chromosomes were removed by the ‘annotateCNV4’ function before visualization. To validate the results of Copy-scAT, we used another R package, Alleloscope^60^ (version 1.0.1) to infer gains and losses at the chromosome scale. We followed its recommended tutorials for snATAC-seq: SNPs were called in merged bam files by bcftools (version 1.9) and further processed by vatrix (1.1.22). Finally, ‘plot_scATAC_snv’ implemented in Alleloscope was used to illustrate the copy number alterations based on the computed reference allele and alternative allele count matrices with default parameters.

### Correlation analysis of CNV prediction results between snRNA-seq and snATAC-seq

A correlation-based method was adopted to validate the CNV prediction results in two modalities^91^. Gene expression-level and bin-level CNV score matrices generated by inferCNV and Alleloscope were normalized with values scaled to a range from –2 to 2. We calculated the average value of total CNV scores for genes/bins contained in each region and assigned the results to the cytobands (different annotated chromosome regions). The extent of deletion and duplication effects on cytobands was determined as the mean CNV scores defined by malignant pseudobulks minus the mean CNV scores calculated by normal pseudobulks. The cytoband-level CNV score matrix generated by Copy-scAT was scaled to the same range and corrected following the same procedure. Spearman correlation coefficients were then calculated between the corrected CNV scores in inferCNV and Copy-scAT across 42 overlapped cytobands (43 overlapped cytobands for Alleloscope and Copy-scAT). Cytobands were labeled as del effects or dup effects only when results in all the methods (inferCNV, Alleloscope, Copy-scAT) showed the same trend (Supplementary Table S5).

### Infer the changes of GRNs

SCRIP was used to obtain the potential downstream target genes of TFs of interest. We filtered low-quality target genes and enriched possibly influenced pathways using ClusterProfiler with *p*-value and *q*-value setting to 0.1. We identified 21 common regulatory pathways in Gene Ontology’s enrichment results. We further found 49 genes which occurred in single or multiple pathways with higher frequency. Those genes were selected and denoted as key genes. Interaction information of those genes was acquired by online database STRING^92^ and visualized by software Cytoscape (version 3.9)^93^. Outlier proteins were manually removed. For differentially expressed target gene in normal and malignant cells (avg.logFC > 0.25 & BH-adjusted *p*-value < 0.05), the enrichment analysis was performed in a similar way.

### GSVA enrichment

GSVA of RNA-seq data was performed at pseudobulk level. We used the R package msigdbr (version 7.5.1) to download the tumor hallmark gene modules from the MSigDB database. Pseudobulks were classified into three categories before mean gene expression analysis (‘AverageExpression’ module in Seurat). We computed the GSVA enrichment scores of these gene modules by ‘gsva’ function in R package GSVA (version 1.42.0) and the results were visualized by heatmap.

### H&E, IHC and IF stainings

Tumor samples, tumor-adjacent tissue samples and normal lung tissue samples were fixed in 4% paraformaldehyde for 48 h and then embedded in paraffin. Paraffin blocks were cut into 5-μm sections and subjected to HE, IHC, or IF staining.

For IHC, slides were deparaffinized through graded xylenes (45 min) and graded ethanol (20 min), and then antigen retrieval was performed using citrate buffer at pH 6.0 (G1202, Servicebio) for 3 min. Slide were then washed in PBS (pH 7.4) for 15 min with moderate rotation. After washing, slides were blocked with 0.3% H_2_O_2_ (25 min). After PBS washing, slides were then blocked with 3% BSA (30 min), followed by staining with primary antibody at 4 °C overnight (VIM, cat# GB111308; KI67, cat# GB111141; TP63, cat# GB11396-1; Servicebio). After PBS washing, slides were incubated with HRP-conjugated Goat Anti-Rabbit IgG (H + L) (cat# GB23303, Servicebio) at room temperature for 50 min, followed by using Dako REAL™ DAB+ Chromogen and Dako REAL™ Substrate Buffer (cat# K5007, DAKO) to visualize staining signals under light microscopy (cat# XSP-C204, CIC), finally counterstained using hematoxylin solution for 3 min. Stained slides were scanned using MIDI (Pannoramic).

For IF, procedures before primary antibody incubation were the same as IHC, except for antigen retrieval step (EDTA at pH 9.0 for 15 min). Slides were incubated with primary antibody at 4 °C overnight (KI67, cat# GB111141; Servicebio), followed by secondary antibodies. Next, the slides were counterstained with DAPI (DAPI, cat# G1012; Servicebio) for 10 min and then mounted. Images were taken with ECLIPSE C1 confocal microscope (DS-U3, NIKON).

### Supplementary information

Supplementary Figures

Supplementary Methods_Microwell-seq3 Protocols

Supplementary Table S1_Sequences of oligos used in this study

Supplementary Table S2_Differentially expressed genes between Microwell-seq3 and 10X Genomics (single cell, whole brain) in different mouse brain cell types

Supplementary Table S3_Differentially expressed genes between Microwell-seq3 and 10X Genomics (single nuclei) in common mouse brain cell types

Supplementary Table S4_Common shared target genes of critical regulons in different gene ontology pathways of malignant cells

Supplementary Table S5_Comparison of chromosome-level CNV scores between inferCNV, Copy-scAT and Alleloscope

## Data Availability

Raw data are available in Gene Expression Omnibus under accession number GSE225134. Processed data are available at figshare (https://figshare.com/articles/dataset/MW3-Dataset/22154066). In the benchmarking part, we used data from other representative methods and sources as follows: Smart-seq3 (E-MTAB-8735), VASA-plate (GSE176588), FLASH-seq (Sequence Read Archive, PRJNA816486), Smart-seq-total (GSE151334), 10X Genomics (10x Genomics official website), Microwell-seq2 (GSE175413) and Drop-seq (GSE63269), sci-ATAC-seq (mouse tissues) (GSE111586), Mouse Cell Atlas (http://bis.zju.edu.cn/MCA/), 10X Genomics scRNA-seq data of mouse whole brain (GSM6617915), 10X Genomics snRNA-seq of mouse cortex (GSE140511), 10X Genomics snRNA-seq of mouse cerebellum (GSE165371). 10X Genomics snRNA-seq data of mouse heart, kidney and liver are available on the official website.

## Code availability

Code and analysis scripts related to the methods are available in the GitHub repository https://github.com/AritaZ-hang/MW3.
