# Supplementary material for: Fast and flexible profiling of chromatin accessibility and total RNA expression in single nuclei using Microwell-seq3
Source: Cell Discov. 2024 Mar 26;10:33. doi: 10.1038/s41421-023-00642-z (PMC10966074; doi:10.1038/s41421-023-00642-z)
Supplement: Supplementary file 1 — Supplementary Figures [file 41421_2023_642_MOESM1_ESM.pdf]

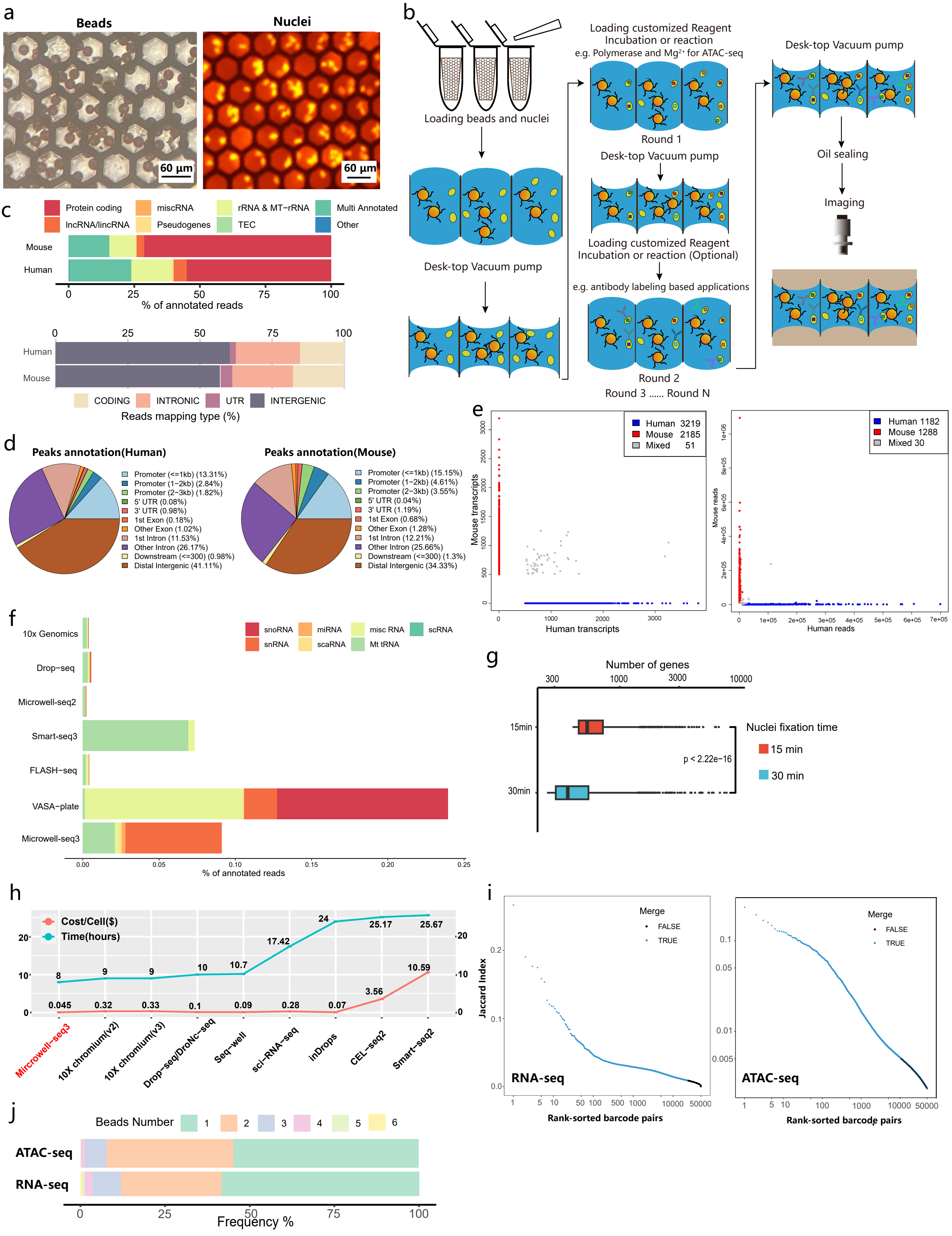
a
Beads
Nuclei
60 µm
60 µm
b
Loading beads and nuclei
Desk-top Vacuum pump
Loading customized Reagent
Incubation or reaction
e.g. Polymerase and Mg2+ for ATAC-seq
Round 1
Desk-top Vacuum pump
Loading customized Reagent
Incubation or reaction (Optional)
e.g. antibody labeling based applications
Round 2
Round 3 ...... Round N
Desk-top Vacuum pump
Oil sealing
Imaging
c
Protein coding
lncRNA/lincRNA
miscRNA
Pseudogenes
rRNA & MT−rRNA
TEC
Multi Annotated
Other
Mouse
Human
% of annotated reads
CODING
INTRONIC
UTR
INTERGENIC
Reads mapping type (%)
d
Peaks annotation(Human)
Promoter (<=1kb) (13.31%)
Promoter (1−2kb) (2.84%)
Promoter (2−3kb) (1.82%)
5' UTR (0.08%)
3' UTR (0.98%)
1st Exon (0.18%)
Other Exon (1.02%)
1st Intron (11.53%)
Other Intron (26.17%)
Downstream (<=300) (0.98%)
Distal Intergenic (41.11%)
Peaks annotation(Mouse)
Promoter (<=1kb) (15.15%)
Promoter (1−2kb) (4.61%)
Promoter (2−3kb) (3.55%)
5' UTR (0.04%)
3' UTR (1.19%)
1st Exon (0.68%)
Other Exon (1.28%)
1st Intron (12.21%)
Other Intron (25.66%)
Downstream (<=300) (1.3%)
Distal Intergenic (34.33%)
e
Human 3219
Mouse 2185
Mixed 51
Mouse transcripts
Human transcripts
Human 1182
Mouse 1288
Mixed 30
Mouse reads
Human reads
f
10x Genomics
Drop−seq
Microwell−seq2
Smart−seq3
FLASH−seq
VASA−plate
Microwell−seq3
snoRNA
snRNA
miRNA
scaRNA
misc RNA
Mt tRNA
scRNA
% of annotated reads
g
Number of genes
15min
30min
p < 2.22e−16
Nuclei fixation time
15 min
30 min
h
Cost/Cell($)
Time(hours)
Microwell−seq3
10X chromium(v2)
10X chromium(v3)
Drop−seq/DroNc−seq
Seq−well
sci−RNA−seq
inDrops
CEL−seq2
Smart−seq2
i
Merge
FALSE
TRUE
Jaccard Index
RNA-seq
Rank-sorted barcode pairs
ATAC-seq
Rank-sorted barcode pairs
j
Beads Number
ATAC-seq
RNA-seq
Frequency %

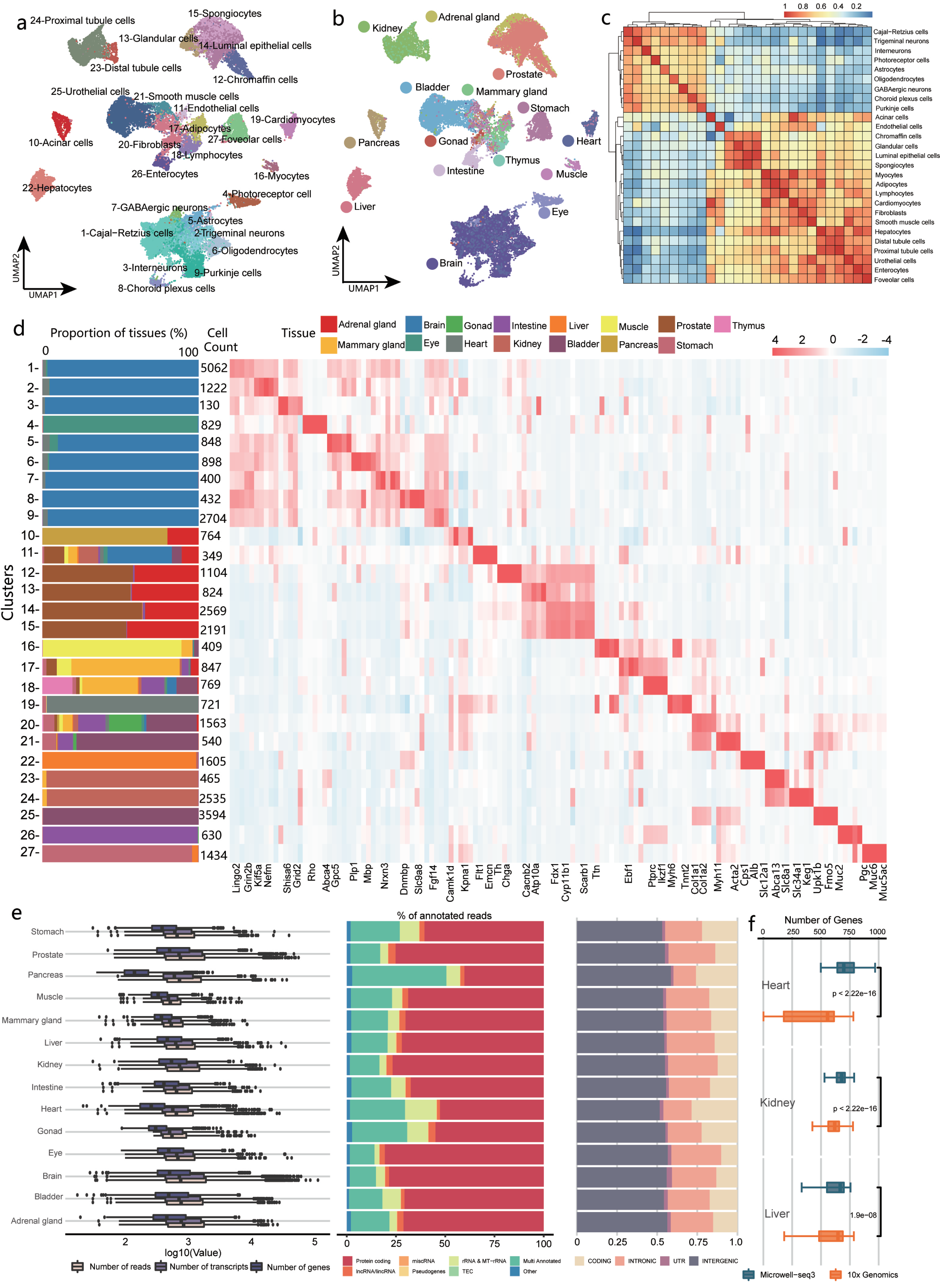

a
24-Proximal tubule cells
15-Spongiocytes
13-Glandular cells
14-Luminal epithelial cells
23-Distal tubule cells
12-Chromaffin cells
25-Urothelial cells
21-Smooth muscle cells
11-Endothelial cells
19-Cardiomyocytes
10-Acinar cells
17-Adipocytes
20-Fibroblasts
27-Foveolar cells
18-Lymphocytes
26-Enterocytes
16-Myocytes
22-Hepatocytes
4-Photoreceptor cell
7-GABAergic neurons
5-Astrocytes
1-Cajal−Retzius cells
2-Trigeminal neurons
6-Oligodendrocytes
3-Interneurons
9-Purkinje cells
8-Choroid plexus cells
UMAP2
UMAP1
b
Adrenal gland
Kidney
Prostate
Bladder
Mammary gland
Stomach
Pancreas
Gonad
Heart
Thymus
Intestine
Muscle
Liver
Eye
Brain
UMAP2
UMAP1
c
1 0.8 0.6 0.4 0.2
Cajal−Retzius cells
Trigeminal neurons
Interneurons
Photoreceptor cells
Astrocytes
Oligodendrocytes
GABAergic neurons
Choroid plexus cells
Purkinje cells
Acinar cells
Endothelial cells
Chromaffin cells
Glandular cells
Luminal epithelial cells
Spongiocytes
Myocytes
Adipocytes
Lymphocytes
Cardiomyocytes
Fibroblasts
Smooth muscle cells
Hepatocytes
Distal tubule cells
Proximal tubule cells
Urothelial cells
Enterocytes
Foveolar cells
d
Proportion of tissues (%)
0
100
Cell Count
Tissue
Adrenal gland
Brain
Gonad
Intestine
Liver
Muscle
Prostate
Thymus
Mammary gland
Eye
Heart
Kidney
Bladder
Pancreas
Stomach
4 2 0 -2 -4
Clusters
1- 5062
2- 1222
3- 130
4- 829
5- 848
6- 898
7- 400
8- 432
9- 2704
10- 764
11- 349
12- 1104
13- 824
14- 2569
15- 2191
16- 409
17- 847
18- 769
19- 721
20- 1563
21- 540
22- 1605
23- 465
24- 2535
25- 3594
26- 630
27- 1434
Lingo2
Grin2b
Kif5a
Nefm
Shisa6
Grid2
Rho
Abca4
Gpc5
Plp1
Mbp
Nrxn3
Dnmbp
Slc9a8
Fgf14
Camk1d
Kpna1
Flt1
Emcn
Th
Chga
Cacnb2
Atp10a
Fdx1
Cyp11b1
Scarb1
Ttn
Ebf1
Ptprc
Ikzf1
Myh6
Tnnt2
Col1a1
Col1a2
Myh11
Acta2
Cps1
Alb
Slc12a1
Abca13
Slc8a1
Slc34a1
Keg1
Upk1b
Fmo5
Muc2
Pgc
Muc6
Muc5ac
e
Stomach
Prostate
Pancreas
Muscle
Mammary gland
Liver
Kidney
Intestine
Heart
Gonad
Eye
Brain
Bladder
Adrenal gland
2 3 4 5
log10(Value)
Number of reads
Number of transcripts
Number of genes
% of annotated reads
0 25 50 75 100
Protein coding
miscRNA
rRNA & MT−rRNA
Multi Annotated
lncRNA/lincRNA
Pseudogenes
TEC
Other
0 0.25 0.5 0.75 1.0
CODING
INTRONIC
UTR
INTERGENIC
f
Number of Genes
0 250 500 750 1000
Heart
p < 2.22e−16
Kidney
p < 2.22e−16
Liver
1.9e−08
Microwell−seq3
10x Genomics

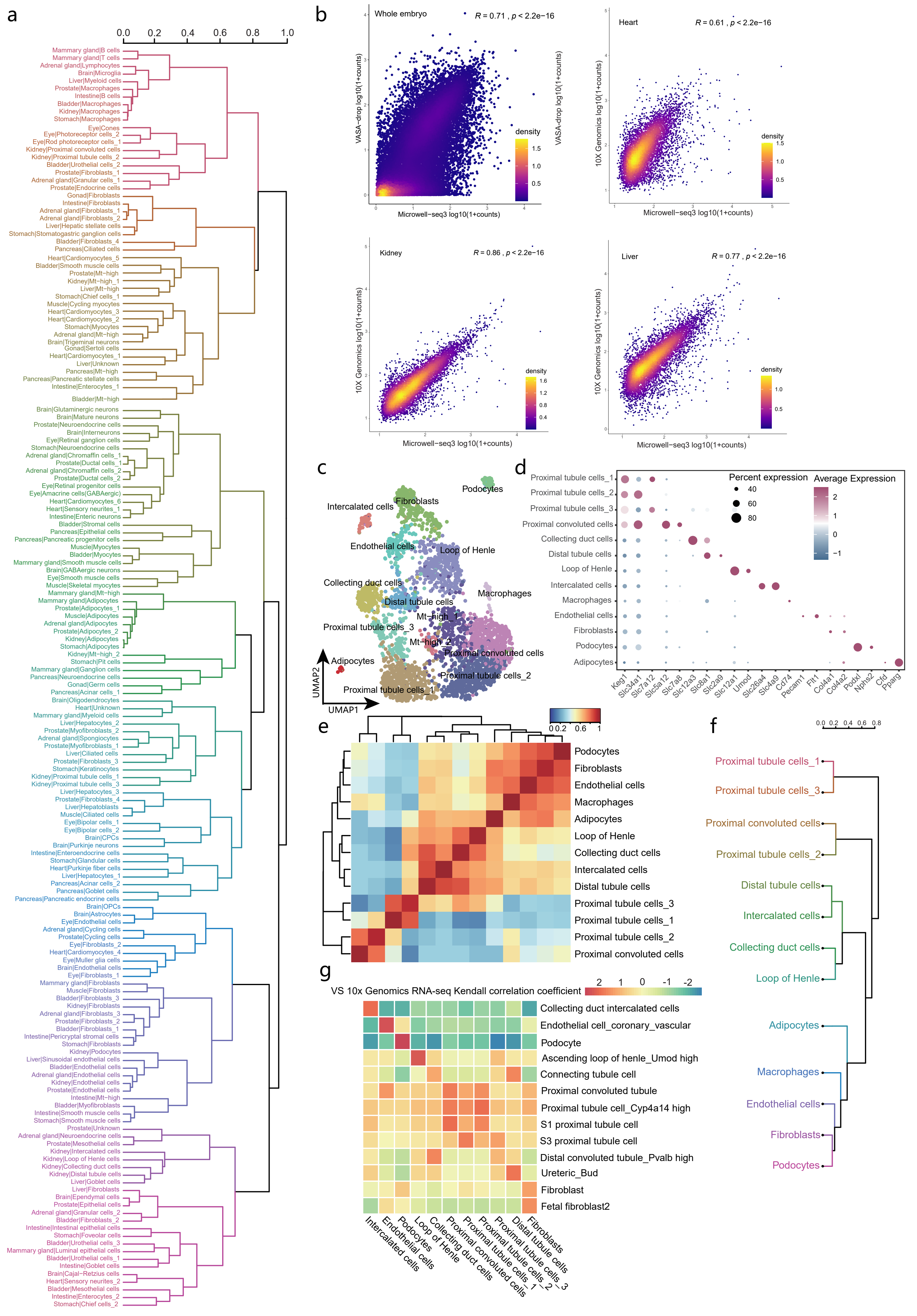

a
0.0 0.2 0.4 0.6 0.8 1.0
Mammary gland|B cells
Mammary gland|T cells
Adrenal gland|Lymphocytes
Brain|Microglia
Liver|Myeloid cells
Prostate|Macrophages
Intestine|B cells
Bladder|Macrophages
Kidney|Macrophages
Stomach|Macrophages
Eye|Cones
Eye|Photoreceptor cells_2
Eye|Rod photoreceptor cells_1
Kidney|Proximal convoluted cells
Kidney|Proximal tubule cells_2
Bladder|Urothelial cells_2
Prostate|Fibroblasts_1
Adrenal gland|Granular cells_1
Prostate|Endocrine cells
Gonad|Fibroblasts
Intestine|Fibroblasts
Adrenal gland|Fibroblasts_1
Adrenal gland|Fibroblasts_2
Liver|Hepatic stellate cells
Stomach|Stomatogastric ganglion cells
Bladder|Fibroblasts_4
Pancreas|Ciliated cells
Heart|Cardiomyocytes_5
Bladder|Smooth muscle cells
Prostate|Mt−high
Kidney|Mt−high_1
Liver|Mt−high
Stomach|Chief cells_1
Muscle|Cycling myocytes
Heart|Cardiomyocytes_3
Heart|Cardiomyocytes_2
Stomach|Myocytes
Adrenal gland|Mt−high
Brain|Trigeminal neurons
Gonad|Sertoli cells
Heart|Cardiomyocytes_1
Liver|Unknown
Pancreas|Mt−high
Pancreas|Pancreatic stellate cells
Intestine|Enterocytes_1
Bladder|Mt−high
Brain|Glutaminergic neurons
Brain|Mature neurons
Prostate|Neuroendocrine cells
Brain|Interneurons
Eye|Retinal ganglion cells
Stomach|Neuroendocrine cells
Adrenal gland|Chromaffin cells_1
Prostate|Ductal cells_1
Adrenal gland|Chromaffin cells_2
Prostate|Ductal cells_2
Eye|Retinal progenitor cells
Eye|Amacrine cells(GABAergic)
Heart|Cardiomyocytes_6
Heart|Sensory neurites_1
Intestine|Enteric neurons
Bladder|Stromal cells
Pancreas|Epithelial cells
Pancreas|Pancreatic progenitor cells
Muscle|Myocytes
Bladder|Myocytes
Mammary gland|Smooth muscle cells
Brain|GABAergic neurons
Eye|Smooth muscle cells
Muscle|Skeletal myocytes
Mammary gland|Mt−high
Mammary gland|Adipocytes
Prostate|Adipocytes_1
Muscle|Adipocytes
Adrenal gland|Adipocytes
Prostate|Adipocytes_2
Kidney|Adipocytes
Stomach|Adipocytes
Kidney|Mt−high_2
Stomach|Pit cells
Mammary gland|Ganglion cells
Pancreas|Neuroendocrine cells
Gonad|Germ cells
Pancreas|Acinar cells_1
Brain|Oligodendrocytes
Heart|Unknown
Mammary gland|Myeloid cells
Liver|Hepatocytes_2
Prostate|Myofibroblasts_2
Adrenal gland|Spongiocytes
Prostate|Myofibroblasts_1
Liver|Ciliated cells
Prostate|Fibroblasts_3
Stomach|Keratinocytes
Kidney|Proximal tubule cells_1
Kidney|Proximal tubule cells_3
Liver|Hepatocytes_3
Prostate|Fibroblasts_4
Liver|Hepatoblasts
Muscle|Ciliated cells
Eye|Bipolar cells_1
Eye|Bipolar cells_2
Brain|CPCs
Brain|Purkinje neurons
Intestine|Enteroendocrine cells
Stomach|Glandular cells
Heart|Purkinje fiber cells
Liver|Hepatocytes_1
Pancreas|Acinar cells_2
Pancreas|Goblet cells
Pancreas|Pancreatic endocrine cells
Brain|OPCs
Brain|Astrocytes
Eye|Endothelial cells
Adrenal gland|Cycling cells
Prostate|Cycling cells
Eye|Fibroblasts_2
Heart|Cardiomyocytes_4
Eye|Muller glia cells
Brain|Endothelial cells
Eye|Fibroblasts_1
Mammary gland|Fibroblasts
Muscle|Fibroblasts
Bladder|Fibroblasts_3
Kidney|Fibroblasts
Adrenal gland|Fibroblasts_3
Prostate|Fibroblasts_2
Bladder|Fibroblasts_1
Intestine|Pericryptal stromal cells
Stomach|Fibroblasts
Kidney|Podocytes
Liver|Sinusoidal endothelial cells
Bladder|Endothelial cells
Adrenal gland|Endothelial cells
Kidney|Endothelial cells
Prostate|Endothelial cells
Intestine|Mt−high
Bladder|Myofibroblasts
Intestine|Smooth muscle cells
Stomach|Smooth muscle cells
Prostate|Unknown
Adrenal gland|Neuroendocrine cells
Prostate|Mesothelial cells
Kidney|Intercalated cells
Kidney|Loop of Henle cells
Kidney|Collecting duct cells
Kidney|Distal tubule cells
Liver|Goblet cells
Liver|Fibroblasts
Brain|Ependymal cells
Prostate|Epithelial cells
Adrenal gland|Granular cells_2
Bladder|Fibroblasts_2
Intestine|Intestinal epithelial cells
Stomach|Foveolar cells
Bladder|Urothelial cells_3
Mammary gland|Luminal epithelial cells
Bladder|Urothelial cells_1
Intestine|Goblet cells
Brain|Cajal−Retzius cells
Heart|Sensory neurites_2
Bladder|Mesothelial cells
Intestine|Enterocytes_2
Stomach|Chief cells_2
b
Whole embryo
R = 0.71 , p < 2.2e−16
VASA−drop log10(1+counts)
Microwell−seq3 log10(1+counts)
density
1.5
1.0
0.5
Heart
R = 0.61 , p < 2.2e−16
10X Genomics log10(1+counts)
Microwell−seq3 log10(1+counts)
density
1.5
1.0
0.5
Kidney
R = 0.86 , p < 2.2e−16
10X Genomics log10(1+counts)
Microwell−seq3 log10(1+counts)
density
1.6
1.2
0.8
0.4
Liver
R = 0.77 , p < 2.2e−16
10X Genomics log10(1+counts)
Microwell−seq3 log10(1+counts)
density
1.0
0.5
c
Podocytes
Intercalated cells
Fibroblasts
Endothelial cells
Loop of Henle
Collecting duct cells
Macrophages
Distal tubule cells
Mt−high_1
Proximal tubule cells_3
Mt−high_2
Proximal convoluted cells
Adipocytes
Proximal tubule cells_2
Proximal tubule cells_1
UMAP1
UMAP2
d
Proximal tubule cells_1
Proximal tubule cells_2
Proximal tubule cells_3
Proximal convoluted cells
Collecting duct cells
Distal tubule cells
Loop of Henle
Intercalated cells
Macrophages
Endothelial cells
Fibroblasts
Podocytes
Adipocytes
Percent expression
40
60
80
Average Expression
2
1
0
−1
Keg1 Slc34a1 Slc7a12 Slc5a12 Slc7a8 Slc12a3 Slc8a1 Slc2a9 Slc12a1 Umod Slc26a4 Slc4a9 Cd74 Pecam1 Flt1 Col4a1 Col4a2 Podxl Nphs2 Cfd Pparg
e
0 0.2 0.6 1
Podocytes
Fibroblasts
Endothelial cells
Macrophages
Adipocytes
Loop of Henle
Collecting duct cells
Intercalated cells
Distal tubule cells
Proximal tubule cells_3
Proximal tubule cells_1
Proximal tubule cells_2
Proximal convoluted cells
f
0.0 0.2 0.4 0.6 0.8
Proximal tubule cells_1
Proximal tubule cells_3
Proximal convoluted cells
Proximal tubule cells_2
Distal tubule cells
Intercalated cells
Collecting duct cells
Loop of Henle
Adipocytes
Macrophages
Endothelial cells
Fibroblasts
Podocytes
g
VS 10x Genomics RNA-seq Kendall correlation coefficient
2 1 0 -1 -2
Collecting duct intercalated cells
Endothelial cell_coronary_vascular
Podocyte
Ascending loop of henle_Umod high
Connecting tubule cell
Proximal convoluted tubule
Proximal tubule cell_Cyp4a14 high
S1 proximal tubule cell
S3 proximal tubule cell
Distal convoluted tubule_Pvalb high
Ureteric_Bud
Fibroblast
Fetal fibroblast2
Intercalated cells
Endothelial cells
Podocytes
Loop of Henle
Collecting duct cells
Proximal convoluted cells
Proximal tubule cells_1
Proximal tubule cells_2
Distal tubule cells
Fibroblasts
Proximal tubule cells_3

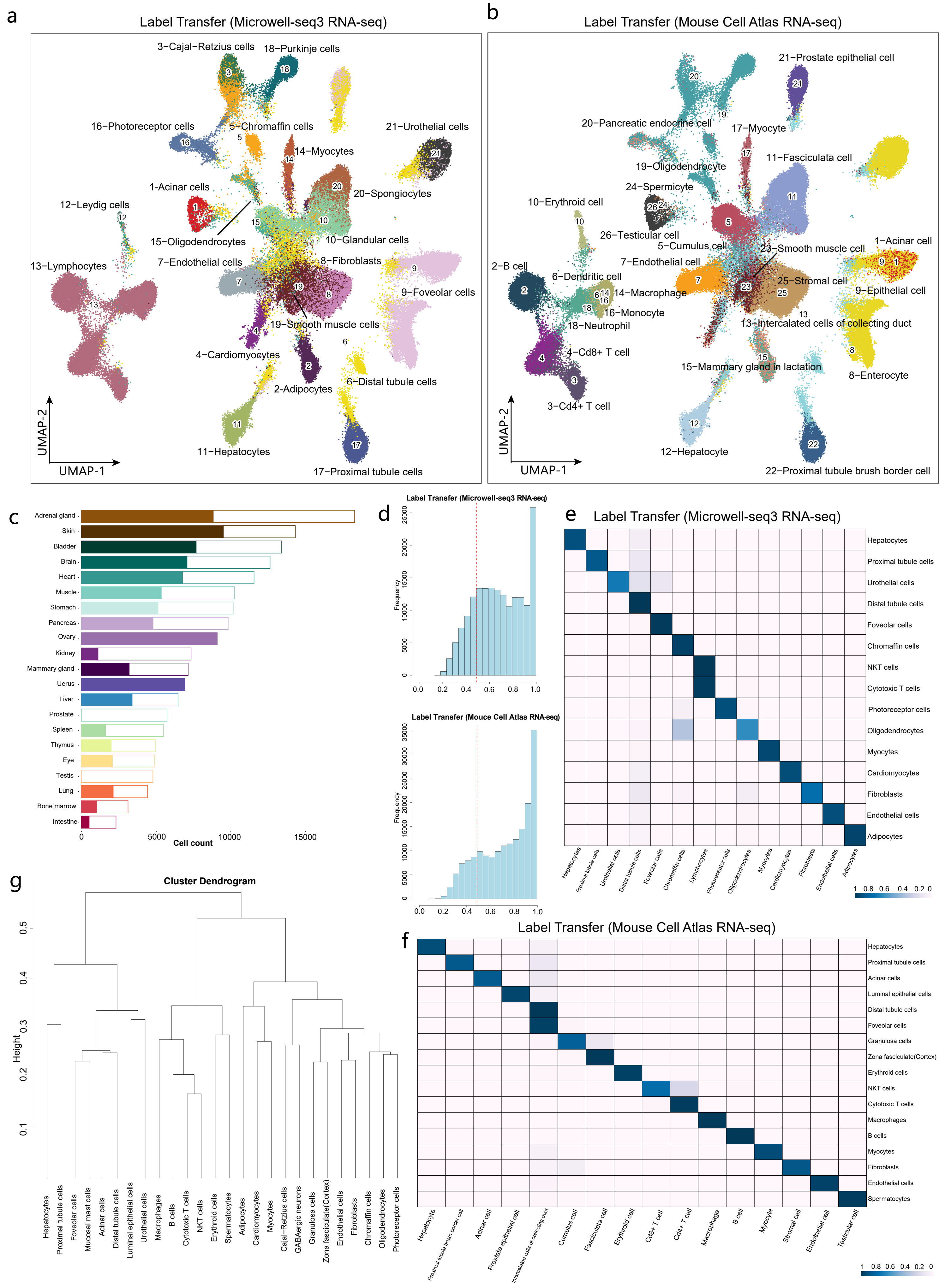

a
Label Transfer (Microwell-seq3 RNA-seq)
3−Cajal−Retzius cells
18−Purkinje cells
16−Photoreceptor cells
5−Chromaffin cells
21−Urothelial cells
14−Myocytes
1-Acinar cells
20−Spongiocytes
12−Leydig cells
15−Oligodendrocytes
10−Glandular cells
13−Lymphocytes
7−Endothelial cells
8−Fibroblasts
9−Foveolar cells
19−Smooth muscle cells
4−Cardiomyocytes
6−Distal tubule cells
2-Adipocytes
11−Hepatocytes
17−Proximal tubule cells
UMAP-2
UMAP-1
b
Label Transfer (Mouse Cell Atlas RNA-seq)
21−Prostate epithelial cell
20−Pancreatic endocrine cell
17−Myocyte
11−Fasciculata cell
19−Oligodendrocyte
24−Spermicyte
10−Erythroid cell
26−Testicular cell
5−Cumulus cell
1−Acinar cell
23−Smooth muscle cell
2−B cell
7−Endothelial cell
6−Dendritic cell
25−Stromal cell
9−Epithelial cell
14−Macrophage
16−Monocyte
18−Neutrophil
13−Intercalated cells of collecting duct
4−Cd8+ T cell
15−Mammary gland in lactation
8−Enterocyte
3−Cd4+ T cell
12−Hepatocyte
22−Proximal tubule brush border cell
c
Adrenal gland
Skin
Bladder
Brain
Heart
Muscle
Stomach
Pancreas
Ovary
Kidney
Mammary gland
Uerus
Liver
Prostate
Spleen
Thymus
Eye
Testis
Lung
Bone marrow
Intestine
0
5000
10000
15000
Cell count
d
Label Transfer (Microwell-seq3 RNA-seq)
Frequency
Label Transfer (Mouce Cell Atlas RNA-seq)
e
Label Transfer (Microwell-seq3 RNA-seq)
Hepatocytes
Proximal tubule cells
Urothelial cells
Distal tubule cells
Foveolar cells
Chromaffin cells
NKT cells
Cytotoxic T cells
Photoreceptor cells
Oligodendrocytes
Myocytes
Cardiomyocytes
Fibroblasts
Endothelial cells
Adipocytes
Lymphocytes
f
Label Transfer (Mouse Cell Atlas RNA-seq)
Hepatocytes
Proximal tubule cells
Acinar cells
Luminal epithelial cells
Distal tubule cells
Foveolar cells
Granulosa cells
Zona fasciculate(Cortex)
Erythroid cells
NKT cells
Cytotoxic T cells
Macrophages
B cells
Myocytes
Fibroblasts
Endothelial cells
Spermatocytes
Hepatocyte
Proximal tubule brush border cell
Acinar cell
Prostate epithelial cell
Intercalated cells of collecting duct
Cumulus cell
Fasciculata cell
Erythroid cell
Cd8+ T cell
Cd4+ T cell
Macrophage
B cell
Myocyte
Stromal cell
Endothelial cell
Testicular cell
g
Cluster Dendrogram
Height
Hepatocytes
Proximal tubule cells
Foveolar cells
Mucosal mast cells
Acinar cells
Distal tubule cells
Luminal epithelial cells
Urothelial cells
Macrophages
B cells
Cytotoxic T cells
NKT cells
Erythroid cells
Spermatocytes
Adipocytes
Cardiomyocytes
Myocytes
Cajal−Retzius cells
GABAergic neurons
Granulosa cells
Zona fasciculate(Cortex)
Endothelial cells
Fibroblasts
Chromaffin cells
Oligodendrocytes
Photoreceptor cells

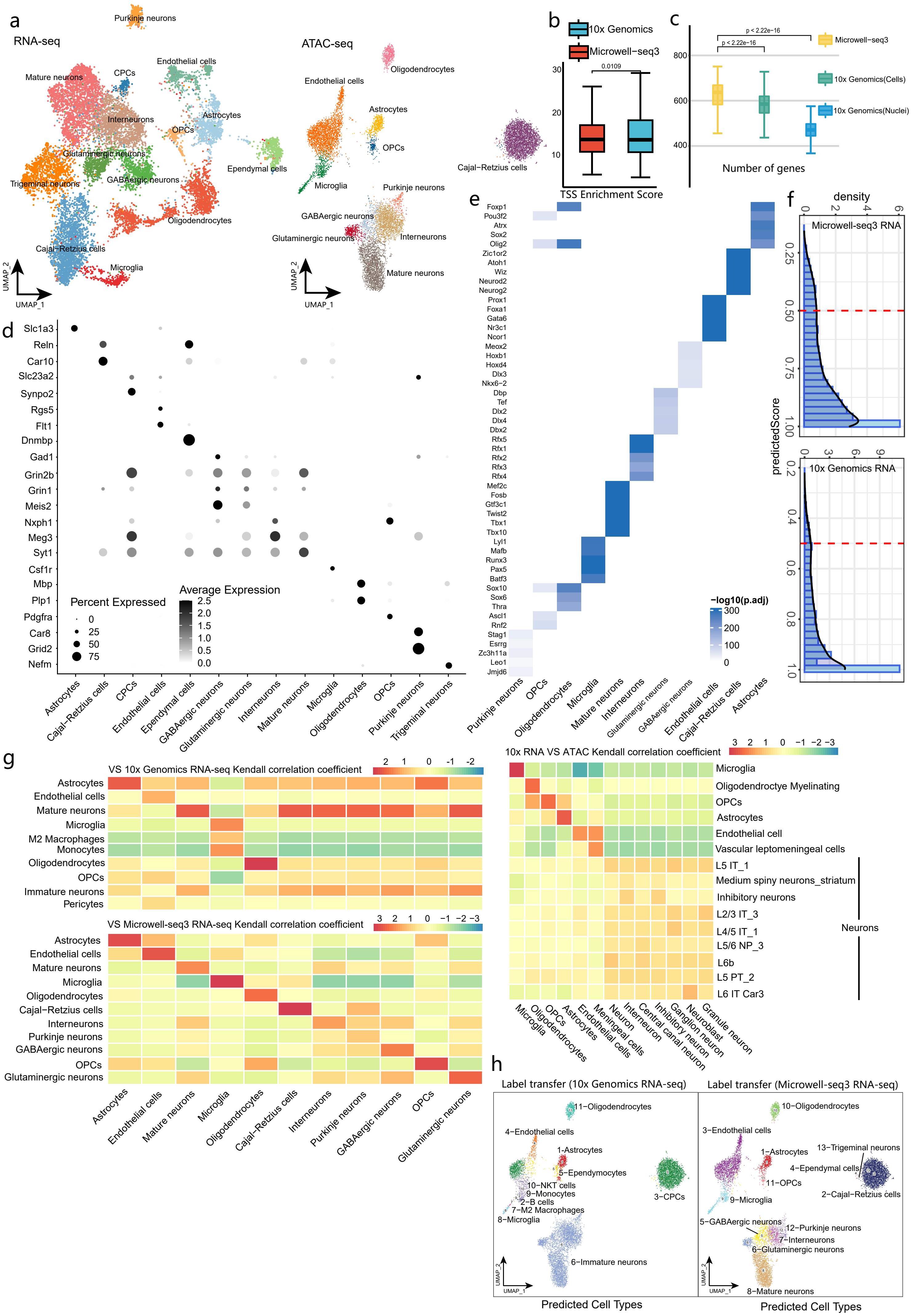

a
RNA-seq
Purkinje neurons
Mature neurons
CPCs
Endothelial cells
Interneurons
OPCs
Astrocytes
Glutaminergic neurons
Ependymal cells
Trigeminal neurons
GABAergic neurons
Oligodendrocytes
Cajal-Retzius cells
Microglia
UMAP_2
UMAP_1
ATAC-seq
Oligodendrocytes
Endothelial cells
Astrocytes
OPCs
Microglia
Purkinje neurons
GABAergic neurons
Glutaminergic neurons
Interneurons
Mature neurons
Cajal-Retzius cells
b
10x Genomics
Microwell-seq3
0.0109
TSS Enrichment Score
c
p < 2.22e-16
p < 2.22e-16
Microwell-seq3
10x Genomics(Cells)
10x Genomics(Nuclei)
Number of genes
d
Slc1a3
Reln
Car10
Slc23a2
Synpo2
Rgs5
Flt1
Dnmbp
Gad1
Grin2b
Grin1
Meis2
Nxph1
Meg3
Syt1
Csf1r
Mbp
Plp1
Pdgfra
Car8
Grid2
Nefm
Percent Expressed
Average Expression
Astrocytes
Cajal-Retzius cells
CPCs
Endothelial cells
Ependymal cells
GABAergic neurons
Glutaminergic neurons
Interneurons
Mature neurons
Microglia
Oligodendrocytes
OPCs
Purkinje neurons
Trigeminal neurons
e
Foxp1
Pou3f2
Atrx
Sox2
Olig2
Zic1or2
Atoh1
Wiz
Neurod2
Neurog2
Prox1
Foxa1
Gata6
Nr3c1
Ncor1
Meox2
Hoxb1
Hoxd4
Dlx3
Nkx6-2
Dbp
Tef
Dlx2
Dlx4
Dbx2
Rfx5
Rfx1
Rfx2
Rfx3
Rfx4
Mef2c
Fosb
Gtf3c1
Twist2
Tbx1
Tbx10
Lyl1
Mafb
Runx3
Pax5
Batf3
Sox10
Sox6
Thra
Ascl1
Rnf2
Stag1
Esrrg
Zc3h11a
Leo1
Jmjd6
-log10(p.adj)
Purkinje neurons
OPCs
Oligodendrocytes
Microglia
Mature neurons
Interneurons
Glutaminergic neurons
GABAergic neurons
Endothelial cells
Cajal-Retzius cells
Astrocytes
f
density
Microwell-seq3 RNA
10x Genomics RNA
predictedScore
g
VS 10x Genomics RNA-seq Kendall correlation coefficient
Astrocytes
Endothelial cells
Mature neurons
Microglia
M2 Macrophages
Monocytes
Oligodendrocytes
OPCs
Immature neurons
Pericytes
VS Microwell-seq3 RNA-seq Kendall correlation coefficient
Astrocytes
Endothelial cells
Mature neurons
Microglia
Oligodendrocytes
Cajal-Retzius cells
Interneurons
Purkinje neurons
GABAergic neurons
OPCs
Glutaminergic neurons
Astrocytes
Endothelial cells
Mature neurons
Microglia
Oligodendrocytes
Cajal-Retzius cells
Interneurons
Purkinje neurons
GABAergic neurons
OPCs
Glutaminergic neurons
10x RNA VS ATAC Kendall correlation coefficient
Microglia
Oligodendroctye Myelinating
OPCs
Astrocytes
Endothelial cell
Vascular leptomeningeal cells
L5 IT_1
Medium spiny neurons_striatum
Inhibitory neurons
L2/3 IT_3
L4/5 IT_1
L5/6 NP_3
L6b
L5 PT_2
L6 IT Car3
Neurons
Microglia
Oligodendrocytes
OPCs
Astrocytes
Endothelial cells
Meningeal cells
Neuron
Interneuron
Central canal neuron
Inhibitory neuron
Ganglion neuron
Neuroblast
Granule neuron
h
Label transfer (10x Genomics RNA-seq)
11-Oligodendrocytes
4-Endothelial cells
1-Astrocytes
5-Ependymocytes
3-CPCs
10-NKT cells
9-Monocytes
2-B cells
7-M2 Macrophages
8-Microglia
6-Immature neurons
Predicted Cell Types
Label transfer (Microwell-seq3 RNA-seq)
10-Oligodendrocytes
3-Endothelial cells
1-Astrocytes
13-Trigeminal neurons
4-Ependymal cells
11-OPCs
9-Microglia
2-Cajal-Retzius cells
5-GABAergic neurons
12-Purkinje neurons
7-Interneurons
6-Glutaminergic neurons
8-Mature neurons
Predicted Cell Types

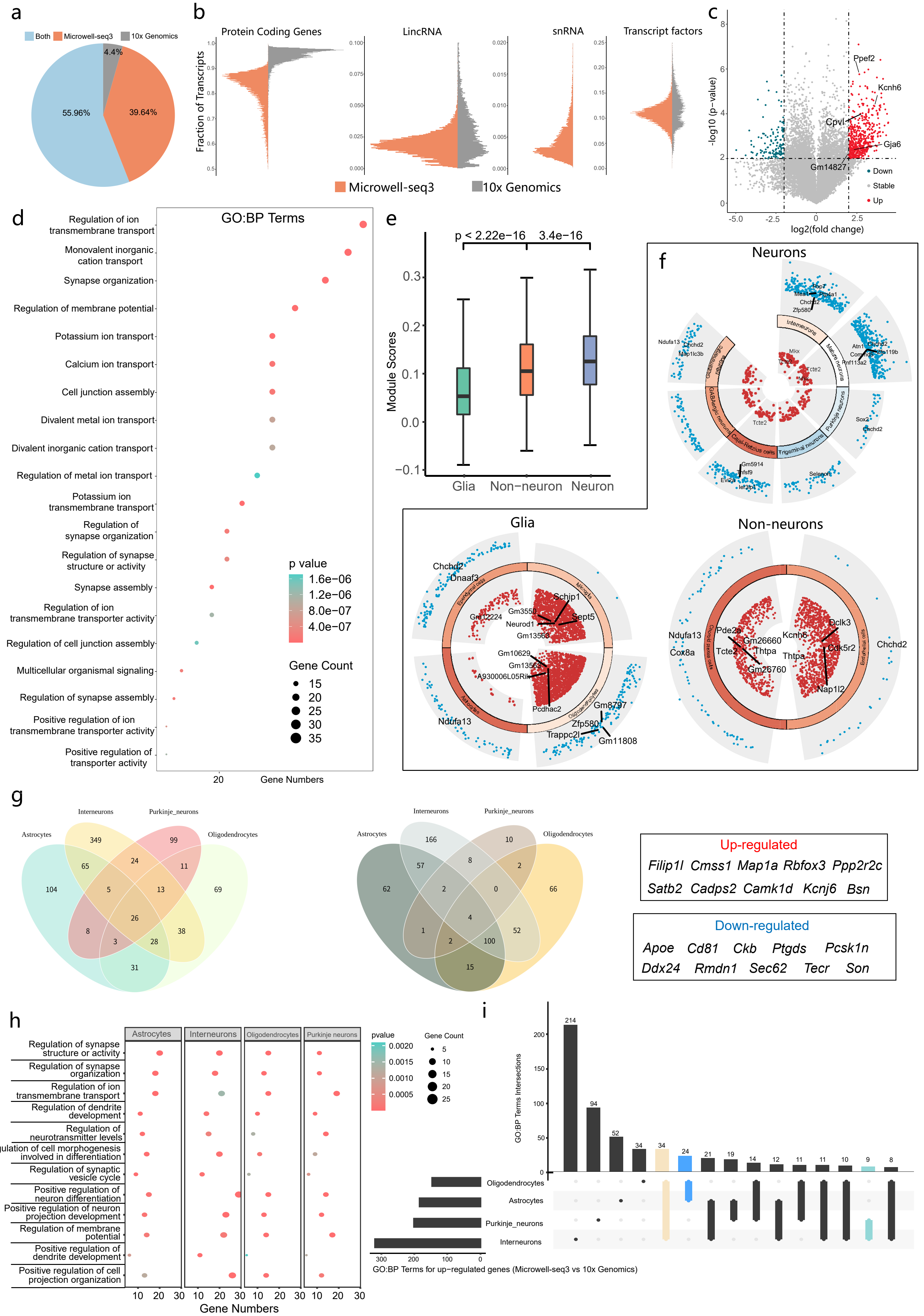

a
Both
Microwell-seq3
10x Genomics
4.4%
55.96%
39.64%
b
Protein Coding Genes
LincRNA
snRNA
Transcript factors
Fraction of Transcripts
Microwell-seq3
10x Genomics
c
Ppef2
Kcnh6
Cpvl
Gja6
Gm14827
-log10 (p-value)
log2(fold change)
Down
Stable
Up
d
GO:BP Terms
Regulation of ion transmembrane transport
Monovalent inorganic cation transport
Synapse organization
Regulation of membrane potential
Potassium ion transport
Calcium ion transport
Cell junction assembly
Divalent metal ion transport
Divalent inorganic cation transport
Regulation of metal ion transport
Potassium ion transmembrane transport
Regulation of synapse organization
Regulation of synapse structure or activity
Synapse assembly
Regulation of ion transmembrane transporter activity
Regulation of cell junction assembly
Multicellular organismal signaling
Regulation of synapse assembly
Positive regulation of ion transmembrane transporter activity
Positive regulation of transporter activity
p value
1.6e−06
1.2e−06
8.0e−07
4.0e−07
Gene Count
15
20
25
30
35
20
Gene Numbers
e
p < 2.22e−16
3.4e−16
Module Scores
Glia
Non−neuron
Neuron
f
Neurons
Glia
Non-neurons
Chchd2
Dnaaf3
Schip1
Sept5
Gm3550
Neurod1
Gm13563
Gm12224
Gm10629
A930006L05Rik
Pcdhac2
Gm8797
Zfp580
Trappc2l
Gm11808
Ndufa13
Pde2a
Gm26660
Tcte2
Thtpa
Gm26760
Kcnh6
Dclk3
Cdk5r2
Nap1l2
Cox8a
g
Astrocytes
Interneurons
Purkinje_neurons
Oligodendrocytes
Up-regulated
Filip1l Cmss1 Map1a Rbfox3 Ppp2r2c
Satb2 Cadps2 Camk1d Kcnj6 Bsn
Down-regulated
Apoe Cd81 Ckb Ptgds Pcsk1n
Ddx24 Rmdn1 Sec62 Tecr Son
h
Astrocytes
Interneurons
Oligodendrocytes
Purkinje neurons
Regulation of synapse structure or activity
Regulation of synapse organization
Regulation of ion transmembrane transport
Regulation of dendrite development
Regulation of neurotransmitter levels
Regulation of cell morphogenesis involved in differentiation
Regulation of synaptic vesicle cycle
Positive regulation of neuron differentiation
Positive regulation of neuron projection development
Regulation of membrane potential
Positive regulation of dendrite development
Positive regulation of cell projection organization
pvalue
0.0020
0.0015
0.0010
0.0005
Gene Count
Gene Numbers
i
GO:BP Terms Intersections
214
94
52
34
34
24
21
19
14
12
11
11
10
9
8
Oligodendrocytes
Astrocytes
Purkinje_neurons
Interneurons
GO:BP Terms for up−regulated genes (Microwell-seq3 vs 10x Genomics)

a

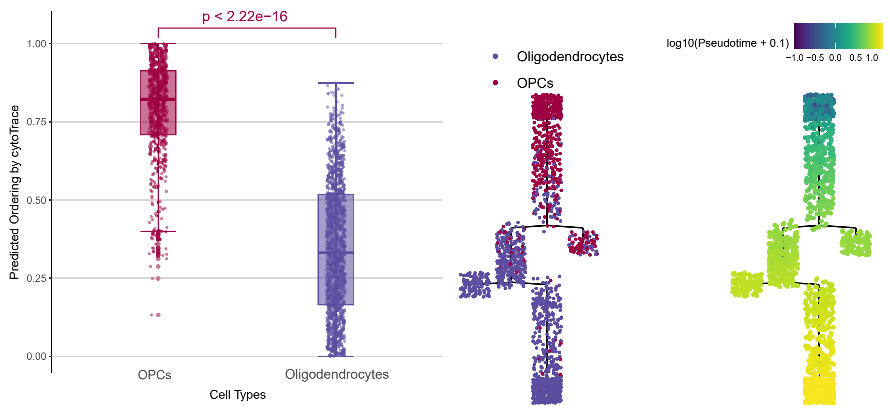
p < 2.22e−16
Predicted Ordering by cytoTrace
1.00
0.75
0.50
0.25
0.00
OPCs
Oligodendrocytes
Cell Types
Oligodendrocytes
OPCs
log10(Pseudotime + 0.1)
−1.0 −0.5 0.0 0.5 1.0

b

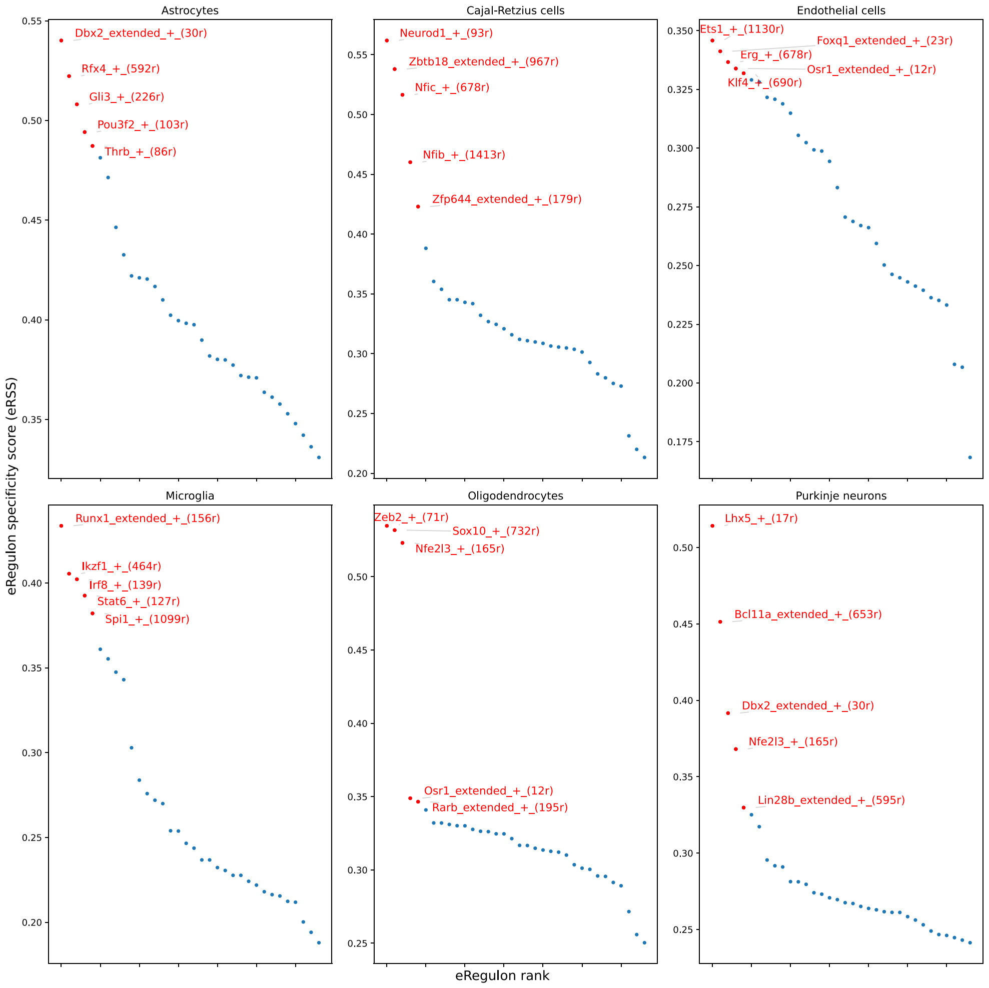
eRegulon specificity score (eRSS)
eRegulon rank
Astrocytes
Dbx2_extended_+_(30r)
Rfx4_+_(592r)
Gli3_+_(226r)
Pou3f2_+_(103r)
Thrb_+_(86r)
Cajal-Retzius cells
Neurod1_+_(93r)
Zbtb18_extended_+_(967r)
Nfic_+_(678r)
Nfib_+_(1413r)
Zfp644_extended_+_(179r)
Endothelial cells
Ets1_+_(1130r)
Foxq1_extended_+_(23r)
Erg_+_(678r)
Osr1_extended_+_(12r)
Klf4_+_(690r)
Microglia
Runx1_extended_+_(156r)
Ikzf1_+_(464r)
Irf8_+_(139r)
Stat6_+_(127r)
Spi1_+_(1099r)
Oligodendrocytes
Zeb2_+_(71r)
Sox10_+_(732r)
Nfe2l3_+_(165r)
Osr1_extended_+_(12r)
Rarb_extended_+_(195r)
Purkinje neurons
Lhx5_+_(17r)
Bcl11a_extended_+_(653r)
Dbx2_extended_+_(30r)
Nfe2l3_+_(165r)
Lin28b_extended_+_(595r)

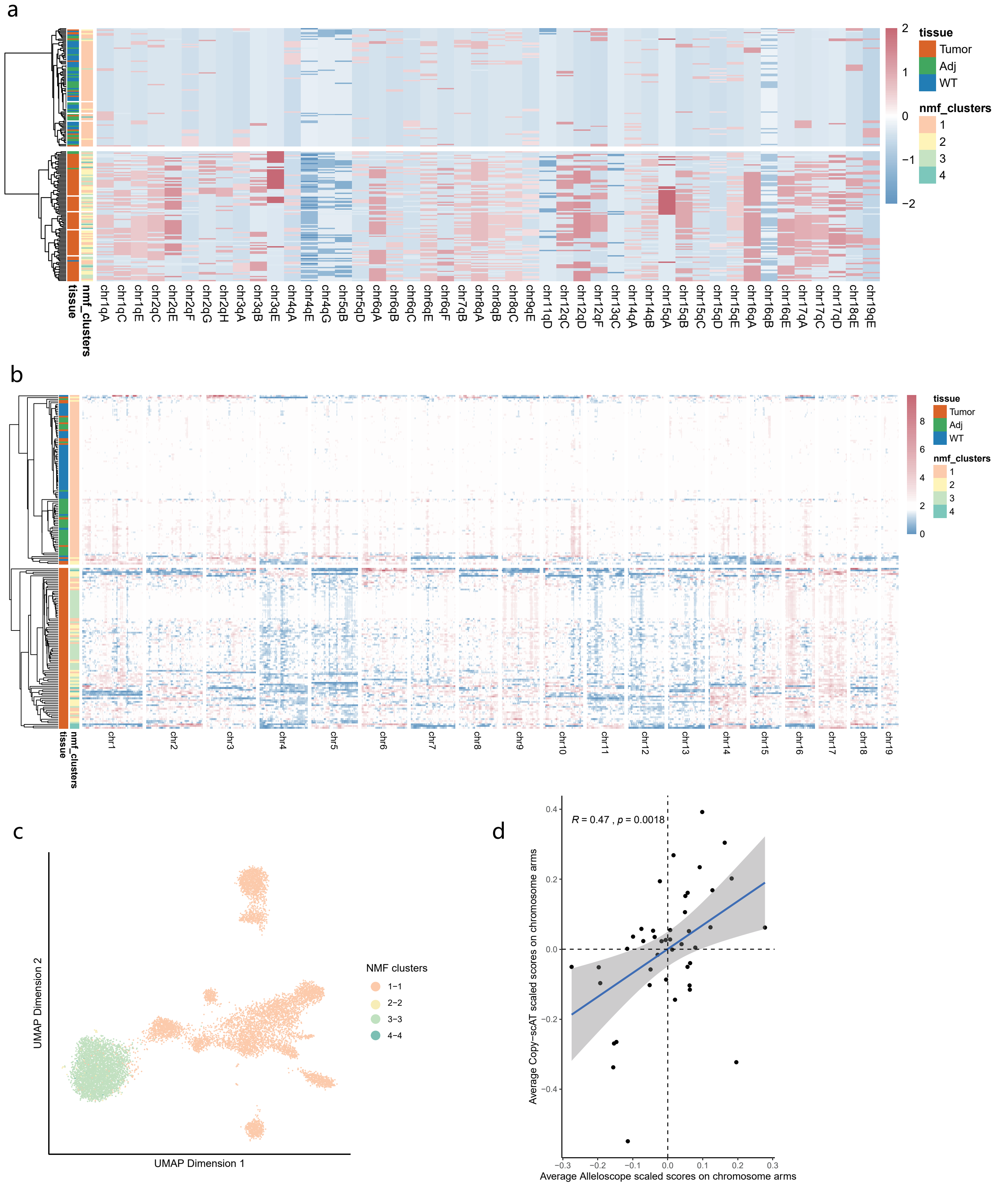
a
tissue
Tumor
Adj
WT
nmf_clusters
1
2
3
4
2
1
0
−1
−2
tissue
nmf_clusters
chr1qA chr1qC chr1qE chr2qC chr2qE chr2qF chr2qG chr2qH chr3qA chr3qB chr3qE chr4qA chr4qE chr4qG chr5qB chr5qD chr6qA chr6qB chr6qC chr6qE chr6qF chr7qB chr8qA chr8qB chr8qC chr9qE chr11qD chr12qC chr12qD chr12qF chr13qC chr14qA chr14qB chr15qA chr15qB chr15qC chr15qD chr15qE chr16qA chr16qB chr16qE chr17qA chr17qC chr17qD chr18qE chr19qE
b
tissue
Tumor
Adj
WT
nmf_clusters
1
2
3
4
8
6
4
2
0
tissue
nmf_clusters
chr1 chr2 chr3 chr4 chr5 chr6 chr7 chr8 chr9 chr10 chr11 chr12 chr13 chr14 chr15 chr16 chr17 chr18 chr19
c
UMAP Dimension 2
UMAP Dimension 1
NMF clusters
1−1
2−2
3−3
4−4
d
R = 0.47 , p = 0.0018
Average Copy−scAT scaled scores on chromosome arms
Average Alleloscope scaled scores on chromosome arms
0.4
0.2
0.0
−0.2
−0.4
−0.3
−0.2
−0.1
0.0
0.1
0.2
0.3

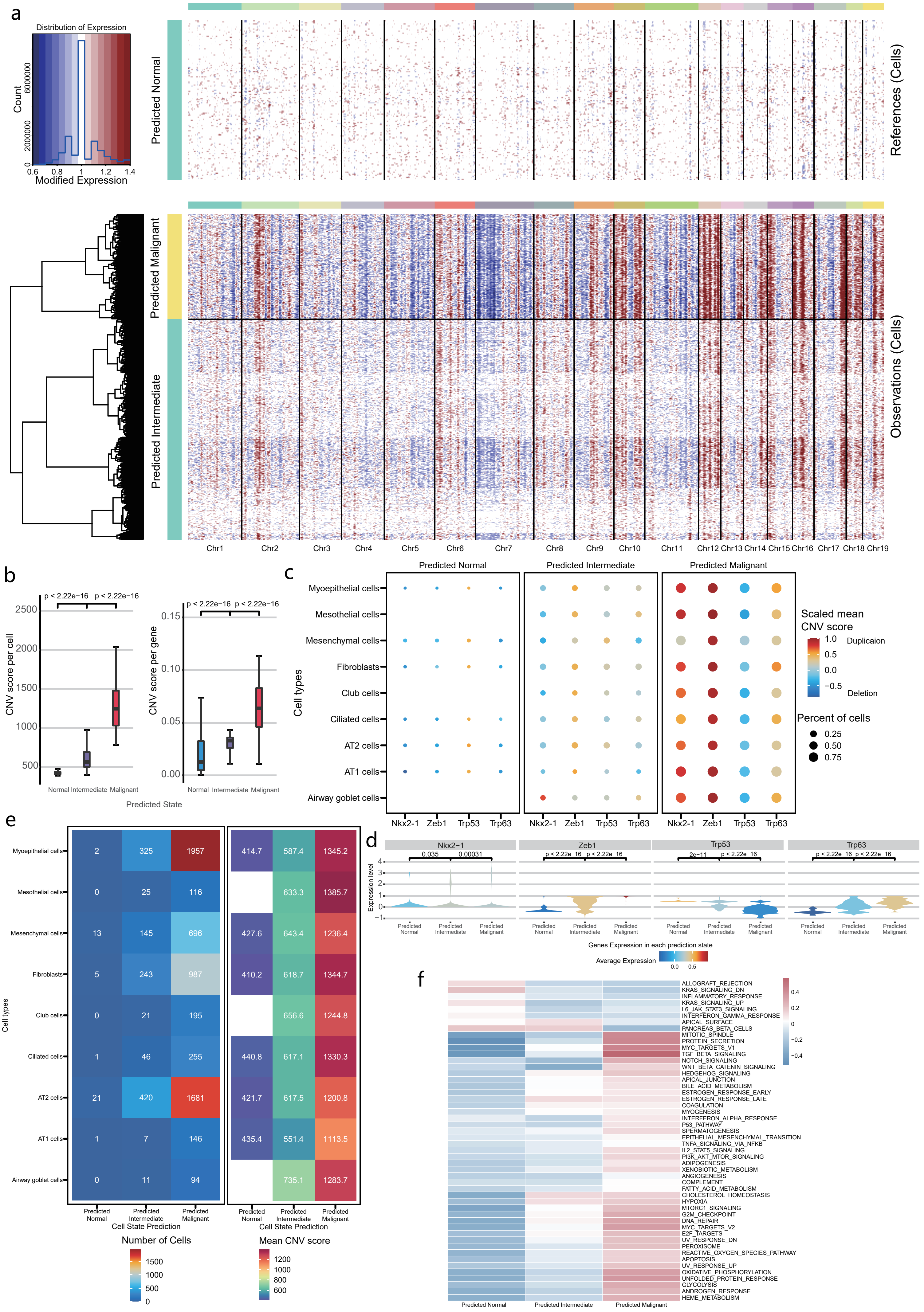
a
Distribution of Expression
Count
Modified Expression
Predicted Normal
References (Cells)
Predicted Malignant
Predicted Intermediate
Observations (Cells)
Chr1 Chr2 Chr3 Chr4 Chr5 Chr6 Chr7 Chr8 Chr9 Chr10 Chr11 Chr12 Chr13 Chr14 Chr15 Chr16 Chr17 Chr18 Chr19
b
p < 2.22e−16 p < 2.22e−16
CNV score per cell
CNV score per gene
Normal Intermediate Malignant
Predicted State
c
Predicted Normal
Predicted Intermediate
Predicted Malignant
Myoepithelial cells
Mesothelial cells
Mesenchymal cells
Fibroblasts
Club cells
Ciliated cells
AT2 cells
AT1 cells
Airway goblet cells
Cell types
Nkx2-1 Zeb1 Trp53 Trp63
Scaled mean CNV score
Duplicaion
Deletion
Percent of cells
0.25
0.50
0.75
d
Nkx2−1 Zeb1 Trp53 Trp63
0.035 0.00031
Expression level
Predicted Normal
Predicted Intermediate
Predicted Malignant
Genes Expression in each prediction state
Average Expression
e
Myoepithelial cells 2 325 1957 414.7 587.4 1345.2
Mesothelial cells 0 25 116 633.3 1385.7
Mesenchymal cells 13 145 696 427.6 643.4 1236.4
Fibroblasts 5 243 987 410.2 618.7 1344.7
Club cells 0 21 195 656.6 1244.8
Ciliated cells 1 46 255 440.8 617.1 1330.3
AT2 cells 21 420 1681 421.7 617.5 1200.8
AT1 cells 1 7 146 435.4 551.4 1113.5
Airway goblet cells 0 11 94 735.1 1283.7
Cell types
Cell State Prediction
Number of Cells
Mean CNV score
f
ALLOGRAFT_REJECTION
KRAS_SIGNALING_DN
INFLAMMATORY_RESPONSE
KRAS_SIGNALING_UP
IL6_JAK_STAT3_SIGNALING
INTERFERON_GAMMA_RESPONSE
APICAL_SURFACE
PANCREAS_BETA_CELLS
MITOTIC_SPINDLE
PROTEIN_SECRETION
MYC_TARGETS_V1
TGF_BETA_SIGNALING
NOTCH_SIGNALING
WNT_BETA_CATENIN_SIGNALING
HEDGEHOG_SIGNALING
APICAL_JUNCTION
BILE_ACID_METABOLISM
ESTROGEN_RESPONSE_EARLY
ESTROGEN_RESPONSE_LATE
COAGULATION
MYOGENESIS
INTERFERON_ALPHA_RESPONSE
P53_PATHWAY
SPERMATOGENESIS
EPITHELIAL_MESENCHYMAL_TRANSITION
TNFA_SIGNALING_VIA_NFKB
IL2_STAT5_SIGNALING
PI3K_AKT_MTOR_SIGNALING
ADIPOGENESIS
XENOBIOTIC_METABOLISM
ANGIOGENESIS
COMPLEMENT
FATTY_ACID_METABOLISM
CHOLESTEROL_HOMEOSTASIS
HYPOXIA
MTORC1_SIGNALING
G2M_CHECKPOINT
DNA_REPAIR
MYC_TARGETS_V2
E2F_TARGETS
UV_RESPONSE_DN
PEROXISOME
REACTIVE_OXYGEN_SPECIES_PATHWAY
APOPTOSIS
UV_RESPONSE_UP
OXIDATIVE_PHOSPHORYLATION
UNFOLDED_PROTEIN_RESPONSE
GLYCOLYSIS
ANDROGEN_RESPONSE
HEME_METABOLISM
Predicted Normal Predicted Intermediate Predicted Malignant

Supplementary figures legends

Supplementary Fig. 1. Benchmarking of Microwell-seq3 data. a, Picture showing the beads and nuclei in the Microwell chips. The beads are 20 μm in diameter, and the diagonal length of each microwell is 60 μm (left). Nuclei were labeled using Ultra GelRed. **b,** Schematic view of the Microwell-seq3 workflow showing the multi-step reaction process. **c,** Proportions of mapped reads in each biotype (up) and mapping results of raw reads (below). Reads are trimmed to 100,000 per sample. **d,** Annotation of the peaks in HEK 293T cells (human) and NIH/3T3 cells (mouse) from the Microwell-seq3 ATAC-seq data. **e,** Scatter plot of the results of the species-mixing experiments showing the numbers of transcripts (left) and unique reads (right) from Microwell-seq3 RNA-seq and ATAC-seq data, respectively. **f,** Proportions of small RNA biotypes in HEK 293T cells across the different methods. **g,** Distribution of the numbers of genes from Microwell-seq3 RNA-seq data in NIH/3T3 cells in a shallow sequencing depth based on the different paraformaldehyde fixation time. The statistical test used was a two-sided t test. **h,** Comparison of the time and reagent costs for Microwell-seq3 RNA-seq and other methods. Source data of other RNA-seq methods is available in a systematic comparison study[1]. **i,** Scatter plot showing the Jaccard index score of barcoded bead pairs in the RNA-seq data (left) or ATAC-seq data (right). True paired beads (blue dots) indicated beads that should be merged in the same microwell with high similarity. **j,** Frequency of the number of detected beads in a microwell based on the Jaccard index from the ATAC-seq and RNA-seq data.

Supplementary Fig. 2. Single-cell clustering of gene expression data in multiple mouse tissues. a, UMAP plot of all cells and their clustering results. The annotation of each cluster was based on canonical cell type markers. **b,** UMAP plot of all cells colored by tissue. **c,** Heatmap showing the AUROC scores for gene expression correlations between different RNA clusters calculated using MetaNeighbor[2]. **d,** Heatmap showing the differentially expressed genes in annotated cell types. Significantly differentially expressed genes are highlighted below. **e,** Boxplot showing the Distribution of mapped reads, transcripts and genes in each tissue per cell (left); Proportions of mapped reads in each biotype (middle); mapping results of raw reads (right). **f,** Distribution of detected gene numbers from Microwell-seq3 and 10x Genomics snRNA-seq data in heart, liver and kidney. Reads were down sampled to 1,000 per nucleus. The statistical test used was a two-sided t test.

Supplementary Fig. 3. Cell type and gene expression correlations in representative tissues. a, Dendrogram showing relationships among cell types in different tissues based on gene expression. **b,** Scatter plot showing the Spearman correlation coefficients between different RNA datasets (VASA-seq and 10x Genomics) and the Microwell-seq3 data. The statistical test used was a two-sided t test. **c,** UMAP plot showing the Microwell-seq3 RNA-seq data of mouse kidney. **d,** Dot plot showing the canonical cell type markers in kidney. **e,** Heatmap showing the AUROC scores for gene expression correlations between different kidney cell types. **f,** Dendrogram showing relationships among kidney cell types. **g,** Heatmap showing the scaled Kendall correlation coefficients between Microwell-seq3 and 10x Genomics snRNA-seq kidney data.

Supplementary Fig. 4. Quality control of Microwell-seq3 ATAC-seq data from wild-type mice. a, b. UMAP plot of cell type annotation results based on label transfer using Microwell-seq3 RNA data (a) and Mouse Cell Atlas RNA data (b). **c,** Bar plot showing the numbers of detected cells in different tissues. The solid bars represent female mice, and the hollow bars represent male mice. **d,** Cell frequency vs. label transfer prediction score between Microwell-seq3 RNA data (up), Mouse Cell Atlas RNA data (below) and Microwell-seq3 mouse ATAC-seq data. **e, f.** Heatmap showing the label transfer prediction correlation between Microwell-seq3 RNA data (e), Mouse Cell Atlas RNA data (f) and Microwell-seq3 mouse ATAC-seq data. **g,** Dendrograms generated by hierarchical clustering of Microwell-seq3 ATAC-seq major cell types in mice.

Supplementary Fig. 5. Identification of cell types in mouse whole brain using Microwell-seq3. a, UMAP plot of mouse brain cells in the Microwell-seq3 RNA-seq (left) and ATAC-seq (right) data. **b,** Comparison of the TSS enrichment score in brain data between Microwell-seq3 and 10x Genomics. Reads (fragments) are down sampled to 2,000 fragments per nucleus. The statistical test used was a two-sided t test. **c,** Distribution of detected gene numbers from Microwell-seq3 and 10x Genomics snRNA-seq mouse brain data. Reads were down sampled to 1,000 per nucleus. The statistical test used was a two-sided t test. **d,** Dot plot showing the average expression levels of marker genes in RNA-seq cell types in (a). The size of the dots represents the percentage of positive cells in each cluster. **e,** Heatmap showing the chromatin accessibility of marker genes in Microwell-

seq3 ATAC-seq data from mouse whole brain tissue. The relative changes in adjusted p values (-$\log_{10}$(p.adj)) are shown on the right. **f,** Label transfer prediction score between Microwell-seq3 RNA data (up), 10x Genomics data (below) and Microwell-seq3 mouse brain ATAC-seq data. **g,** Heatmap showing the scaled Kendall correlation coefficients between 10x Genomics RNA-seq data (left, up), Microwell-seq3 RNA-seq data (left, below) and Microwell-seq3 ATAC-seq data of brain; Heatmap showing the scaled Kendall correlation coefficients between 10x Genomics brain RNA-seq and ATAC-seq data (right). **h,** UMAP plot showing the label transfer results of Microwell-seq3 ATAC-seq data using 10x Genomics RNA-seq (left) and Microwell-seq3 RNA-seq (right).

Supplementary Fig. 6. Differentially expressed genes in Microwell-seq3 mouse brain compared with 10x Genomics. a, Pie chart showing the distribution of detected genes between 10x Genomics (RNA-seq) and Microwell-seq3 mouse whole brain data. **b,** Fraction of transcripts per biotype in Microwell-seq3 RNA-seq compared with 10x Genomics mouse whole brain data. **c,** Volcano plot showing the DEGs between Microwell-seq3 RNA-seq and 10x Genomics data. Non-polyadenylated upregulated genes ($\log_2$-Fold Change > 2 and p value < 0.01) are labeled. Downregulated genes in the Microwell-seq3 data ($\log_2$-Fold Change < -2 and p value < 0.01) are marked in blue. Stably expressed genes with no significant changes are marked in gray. **d,** Gene ontology enrichment analysis showing the biological processes of upregulated genes in (c). **e,** Distribution of upregulated DEGs in (c). The expression levels in upregulated gene sets were evaluated using the AddModuleScore() function in Seurat (the statistical test used was a two-sided t test). **f,** Dot plot showing the DEGs in major brain cell types between Microwell-seq3 and 10x Genomics data. DEGs in each cell subset with |$\log_2$ fold change| > 1 and p value < 0.01 were included (red: upregulated genes in Microwell-seq3, blue: downregulated genes in Microwell-seq3). Non-polyadenylated genes are labeled. **g,** Venn plot showing the overlapping of upregulated and downregulated genes in common cell types between Microwell-seq3 snRNA-seq data and 10x Genomics snRNA-seq data. Top differentially expressed genes are shown on the right. **h,** Dot plot showing the upregulated gene functions in four common cell types. **i,** Upset plot showing the overlapping the up-regulated gene function enrichment terms in four common cell types.

Supplementary Fig. 7. Trajectory analysis and cell type-specific regulon activity in brain. a,

Pseudotime analysis of the oligodendrocytes and OPCs showing the early phase gene expression in OPCs. **b,** Rank of regulons in brain cell types based on the regulon specificity score. The enrichment score of a regulon is defined as target region enrichment per cell. In SCENIC+, the AUCell function ranks genes or regions based on the expression or accessibility per cell as input, and the output is a list of regulons ranked by the AUC value (enrichment of eRegulon target regions).

Supplementary Fig. 8. Identification of malignant cells in snATAC-seq data based on CNV prediction. Heatmap showing the predicted CNV results using Copy-scAT (**a**) and Alleloscope (**b**). The chromosome location in Copy-scAT is based on the Cytoband file type of the mouse genome build mm10. **c,** UMAP plot showing the NMF clustering of cells in Fig. 4c, NMF cluster 1 is labeled as predicted normal cells. **d,** CNV prediction correlation of different cytobands (chromosome regions) between Alleloscope and Copy-scAT.

Supplementary Fig. 9. Identification of malignant cells in tumor and tumor-adjacent tissue in snRNA-seq data. a, Heatmap showing the inferCNV results of Microwell-seq3 RNA-seq data. **b,** Cell state prediction based on the CNV score using inferCNV (see methods). The statistical test used was a two-sided t test. **c,** Dot plot showing the scaled mean CNV score indicating the duplication and deletion of representative regulators. Size of the dots indicated the percent of cells across different prediction state. **d,** Gene expression level of representative regulators across different prediction state. The statistical test used was a two-sided t test. **e,** Cell state prediction based on the CNV score (the number of cells in each state across different cell types). Mean CNV scores of predicted cell state are shown on the right. **f,** Heatmap showing the GSVA results of different cell states in myoepithelial cells.

References

1. Ding, J.R. et al. Systematic comparison of single-cell and single-nucleus RNA-sequencing methods (vol 17, pg 891, 2020). *Nat Biotechnol* **38**, 756-756 (2020).
2. Crow, M., Paul, A., Ballouz, S., Huang, Z.J. & Gillis, J. Characterizing the replicability of cell types defined by single cell RNA-sequencing data using MetaNeighbor. *Nat Commun* **9**, 884 (2018).
