# Supplementary material for: Fast and flexible profiling of chromatin accessibility and total RNA expression in single nuclei using Microwell-seq3
Source: Cell Discov. 2024 Mar 26;10:33. doi: 10.1038/s41421-023-00642-z (PMC10966074; doi:10.1038/s41421-023-00642-z)
Supplement: Supplementary file 2 — Supplementary Methods_Microwell-seq3 Protocols [file 41421_2023_642_MOESM2_ESM.docx]

**Protocols for Microwell-seq3**

**Materials and Methods:**

***Beads:***

The 20 µm carboxyl modified magnetic beads (50 mg ml-1) were obtained from SuZhou KBsphere Co., Ltd. (catalog no. MagCOOH-20190911). The barcoded oligonucleotides on the surface of the beads were synthesized by three rounds of split-pool based PCR as described in Microwell-seq 1.0^[^[^1^](#_ENREF_1)^]^.

1. Carboxyl modified magnetic beads were washed and suspended in 0.1 M MES (2-[N-morpholino] ethanesulfonic acid, catalog no. D11267, OKA bio) with 3.08 mg of EDC (1-ethyl-3 (-3-dimethylaminopropyl) carbodiimide hydrochloride, catalog no. C600433, Sangon Biotech). 6.25 µl of beads were distributed into each well of a 96-well plate.
2. 2.5 µl of amino modified beads_primer_A oligos (80uM in 0.1M MES, Supplementary Table 1, Sangon Biotech, HPLC purification) were then added into each well.
3. After vortexing and incubation for 20 minutes at room temperature (25 °C), 0.5 µl mix (Add 6mg of EDC in 100 µl of 0.1M MES) was distributed into each well.
4. After another round of vortexing and incubation for 20 minutes at room temperature (25 °C), 0.5 µl mix (Add 6mg of EDC in 100 µl of 0.1M MES) was distributed into each well.
5. After vortexing and incubation for 80 minutes at ambient temperature, the beads were collected in 1 ml of 0.1M PBS containing 0.02% Tween-20. The beads were then washed two times in 1 mL of Tris-EDTA (pH 8.0).
6. In the second round of split-pool, the beads were washed with Tris-EDTA (pH 8.0) and suspended in water and split into each well of another 96-well plate containing the PCR mix (20 µl for each well):

1x Phanta Master Mix (catalog no. P515-01, Vazyme)

5µM beads_primer_B oligos (Supplementary Table 1, Sangon Biotech)

PCR program was as follows:

94 °C for 5 min

5 cycles of:

94 °C for 15 s

48.8 °C for 4 min

72 °C for 4 min

4 °C hold

1. After PCR reaction, put beads into 95°C water bath for 6 min to remove complementary chains. Beads were separated with magnetic separator and remove the supernatant quickly for 2 times. The third split-pool procedure was the same as the second one:

PCR mix (20 µl for each well):

1x Phanta Master Mix (catalog no. P515-01, Vazyme)

5µM beads_primer_C oligos (Supplementary Table 1, Sangon Biotech, different beads_primer_C oligos were used for RNA-seq and ATAC-seq)

PCR program was as follows:

94 °C for 5 min

48.8 °C for 20 min

72 °C for 20 min

4 °C hold

1. After PCR reaction, put beads into 95°C water bath for 6 min to remove complementary chains. Beads were separated with magnetic separator and remove the supernatant quickly for 2 times.
2. Then, beads were washed with ddH2O and could be stored in TE-TW (10 mM Tris pH 8.0, 1 mM EDTA, 0.01% Tween20) for at least 8 weeks at 4 °C.

***Chips:***

Microwell chips were obtained from Clarity™ Digital PCR system (catalog no. 12007, JN Medsys, agent by Neoline Technology Co., Ltd.), and custom microwell chips were fabricated based on ZZ-Bio Digital PCR System chips (ZHENZHUN Biotechnology Co., Ltd.). Every chip in the 0.2 ml tube contained 10,000 partitions. The total reaction volume in one chip was approximately 15 µl. The diagonal length of a hexagonal partition is 60 µm.


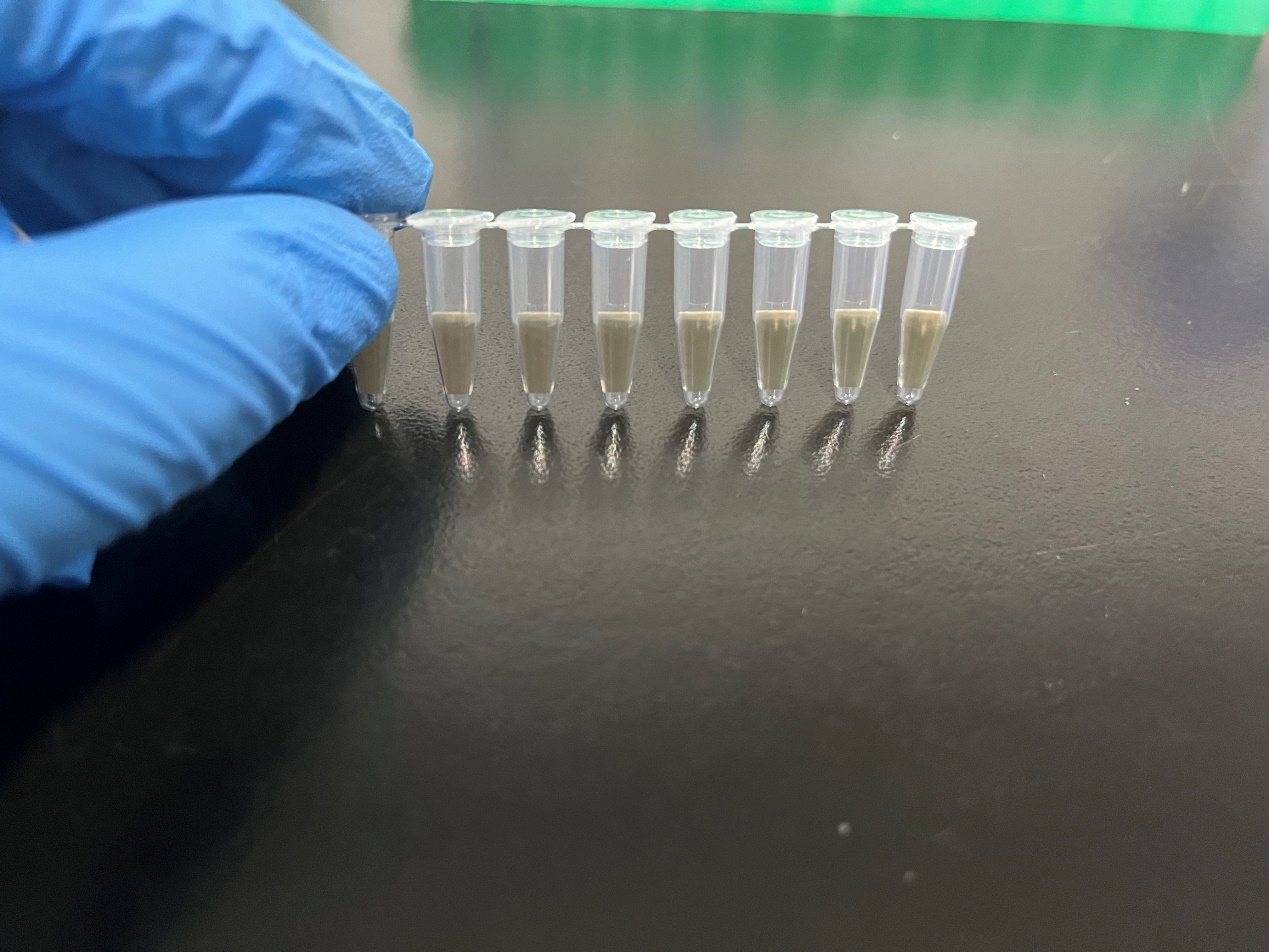


Microwell chips in strips


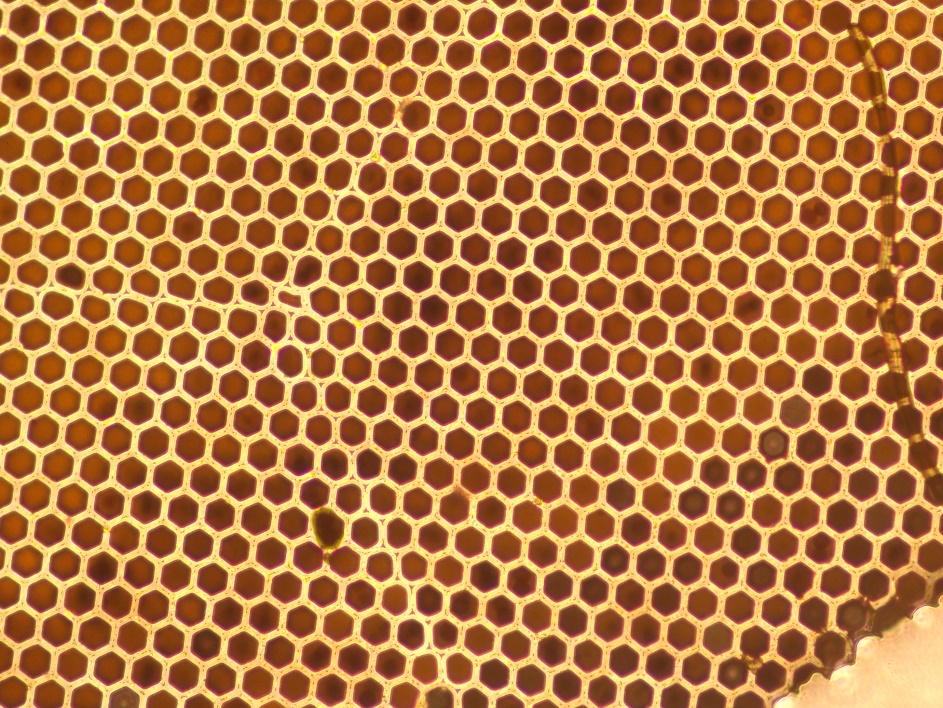


hexagonal partitions with 60 µm diagonal length

***Microwell-seq3 RNA-seq:***

**Nucleus preparation and fixation**

**Cell lines**

For different cell lines, cells were trypsinized and resuspended in cold 1× PBS and pelleted by centrifugation at 300 × g for 5 minutes. The cell pellet was lysed in 1 ml of ice-cold lysis buffer (for RNA assays: 10 mM Tris-HCl (pH 7.4), 10 mM NaCl, 3 mM MgCl2, 0.1% Tween 20, 1% Murine RNase Inhibitor (Vazyme Biotech), 0.1% IGEPAL CA-630, 1 mM DTT; for ATAC-seq: 10 mM Tris-HCl (pH 8.0), 10 mM NaCl, 3 mM MgCl2, 0.1% Tween 20, 0.1% IGEPAL CA-630, 0.01% digitonin (Promega), 1× protease inhibitor (Sangon Biotech)) and incubated on ice for 2 minutes. Then, 5 ml of ice-cold RSBT buffer (10 mM Tris-HCl (pH 7.4), 10 mM NaCl, 3 mM MgCl2, 0.1% Tween 20) was added to terminate the lysis reaction. The medium was then filtered using a 40 μm strainer. Nuclei were centrifuged at 500 × g for 5 min at 4 ℃ and washed with 1 ml of cold RSBT buffer.

**Tissues**

For fresh and frozen tissues, we first cut large tissues (e.g. liver, whole brain, kidney) into small pieces. We recommend using a stainless-steel blender to treat tissues with wide range of sizes. Liquid nitrogen was added and the tissues were ground into homogeneous cold powders. The tissue powders were rapidly transferred into a 1.5-ml centrifuge tube. Around 0.1 ml homogeneous tissue powders could generate at least millions of nuclei. The nuclei isolation step was the same for cell lines and tissues. Isolated nuclei from tissues were centrifuged at 800 × g for 5 minutes in all centrifugation steps.


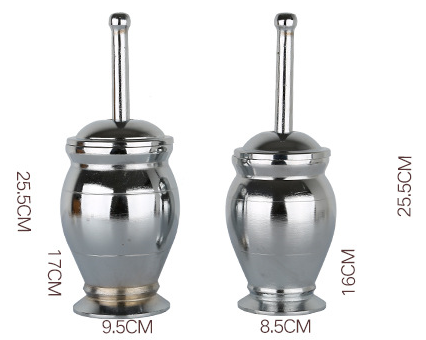


stainless steel blender

**Fixation**

1. In RNA-seq, fresh or frozen nuclei were first resuspended in 100 µl of RSBT buffer (10 mM Tris-HCl (pH 7.4), 10 mM NaCl, 3 mM MgCl2, 0.1% Tween 20), and 10 ml of ice-cold 4% nuclease-free paraformaldehyde was then added carefully.
2. Nuclei were fixed at 4 °C for 15 minutes, and 1.5 ml of 2.5 M glycine (catalog no. A502065-0500, Sangon Biotech) was added to quench the fixation.
3. Fixed nuclei were washed twice with 1 ml of RSBT buffer (by centrifugation at 500 × g for 5 minutes at 4 ℃).

**In situ reverse transcription**

1. Fixed nuclei were counted and then resuspended in reverse transcription buffer mix (each well: 0.5 µl 10 mM dNTPs, 0.5 µl 10% Triton X-100, 1.5 µl PEG8000 (catalog no. 89510, Sigma‒Aldrich), 3.6 µl of fixed nuclei (10,000 nuclei) in RSB buffer without Tween-20) and split into one or more (up to 4) 96-well plates.
2. 1 µl of 10 µM concentration of 384 barcoded random primers (Supplementary Table 1, Sangon Biotech) was added to each well using a multi-channel pipette. The 96-well plates were incubated at 55 ℃ for 5 minutes.
3. The plates were immediately placed on ice. 3 µl of reverse transcription enzyme mix (2 µl of 5× RT buffer (31 mM Tris-HCl (pH 8.0), 37.5 mM NaCl, 3.1 mM MgCl2, 10 mM DTT), 0.5 µl of Murine RNase Inhibitor (catalog no. R301-01, Vazyme), 0.5 µl of Maxima H Minus RTase (catalog no. K1682, Thermo Fisher)) was added into each well.
4. In situ reverse transcription reaction was performed as follows:

10 cycles of:

8 °C for 12 s

15 °C for 30 s

20 °C for 45 s

25 °C for 1 min

30 °C for 1 min

42 °C for 2 min

Final incubation at 42 °C for 30 min (using a 96-well plates rotator placed in the incubator for the final incubation step).

**Exonuclease I treatment**

1. After reverse transcription, the 96-well plates were placed on ice for 2 minutes to stop the reaction. 3 µl of Exonuclease I mix (1.3 µl of 10× Exonuclease I Reaction Buffer (catalog no. M0293L, NEB), 1.5 µl of nuclease-free water, 0.2 µl of Exonuclease I (catalog no. M0293L, NEB)) was added into each well.
2. The plates were slowly rotated at 37 ℃ for 30 minutes in an incubator to perform Exonuclease I treatment of dissociative oligos.
3. After Exonuclease I treatment, nuclei were pooled and washed twice with RSBT buffer.

**Poly(A) tailing**

1. The nuclei were resuspended in poly(A) tailing mix (20 µl of nuclei in RSB buffer, 56 µl of nuclease-free water, 2 µl of Murine RNase Inhibitor (catalog no. R301-01, Vazyme), 10 µl of 10× terminal transferase reaction buffer (catalog no. B0315, NEB), 10 µl of 10× CoCl2 (catalog no. B0252, NEB), 1 µl of 100 mM dATP (catalog no. B500044, Sangon Biotech), 1 µl of Terminal Transferase (catalog no. M0315, NEB))
2. The tube was slowly rotated at 37 ℃ for 15 minutes in an incubator to perform Poly(A) tailing.
3. The nuclei were washed with 1 ml of 3× SSC-T (3× SSC (catalog no. 15557044, Thermo Fisher), 0.05% Tween 20) and twice with 1 ml of RSB buffer. The nuclei were resuspended in RSB buffer before loading on the chips.

**Chip-loading (RNA-seq)**

1. The beads were washed twice with RSB buffer before use. The nuclei and beads were resuspended in loading mix. 20,000 nuclei and 20,000 barcoded beads were loaded onto one chip.
2. The nuclei and beads were resuspended in loading mix, and 11 µl aliquots of loading mix with nuclei and beads (6 µl of nuclease-free water, 5 µl of KAPA HiFi HotStart ReadyMix (catalog no. 07958935001, Roche)) were prepared for one chip.
3. The chip was taken out from the tube using a flat tip tweezers. The loading mix with nuclei and beads was pipetted a few times and quickly added to the chip surface using a flat end pipette tip (note: pipette beads and nuclei several times and quickly add the mixture into the chip to avoid the sinking of beads).


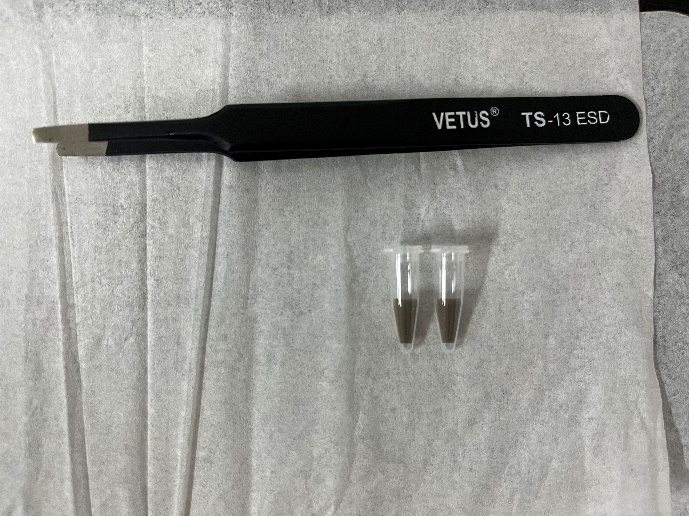


Chips and flat tip tweezers


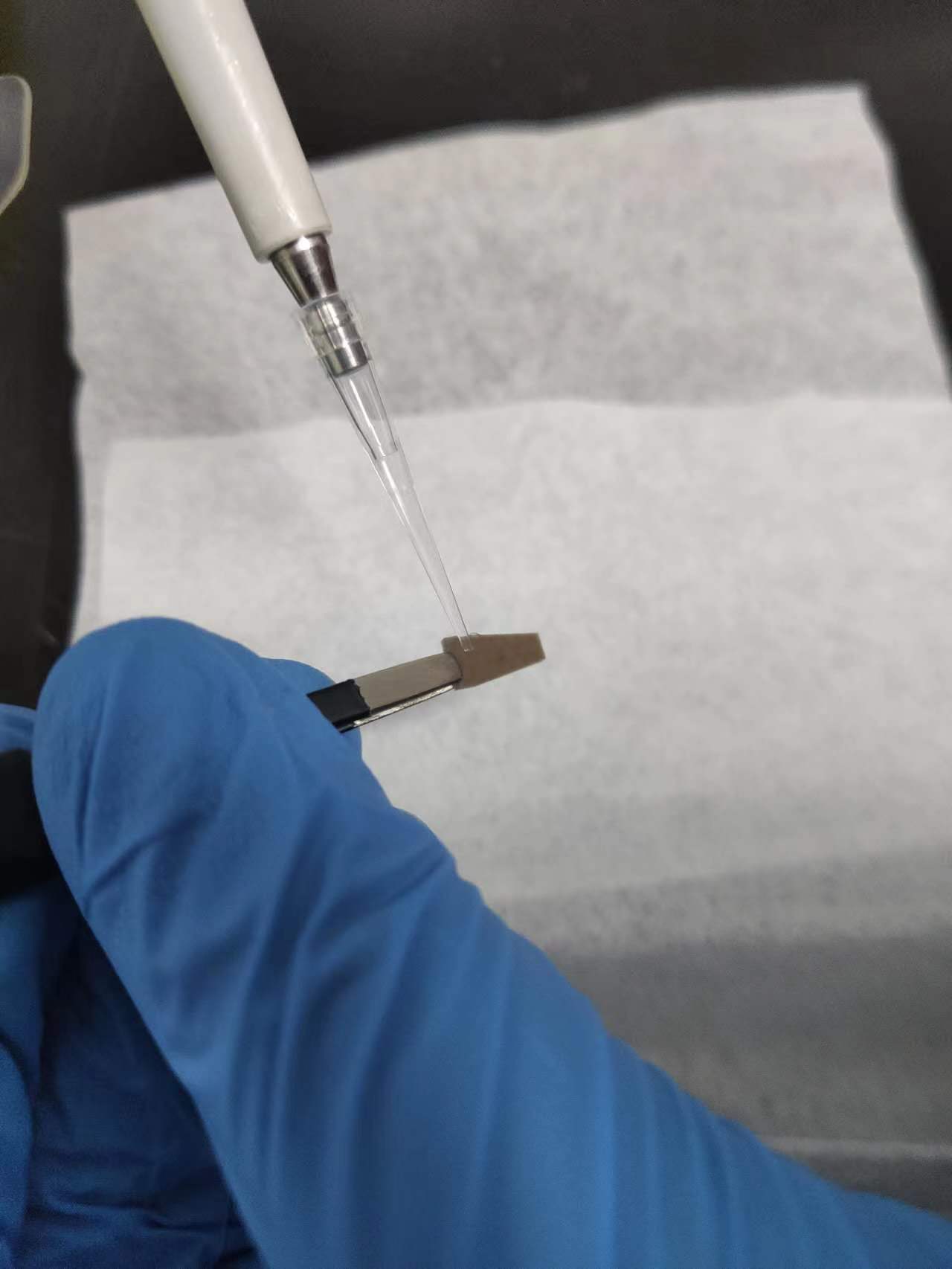

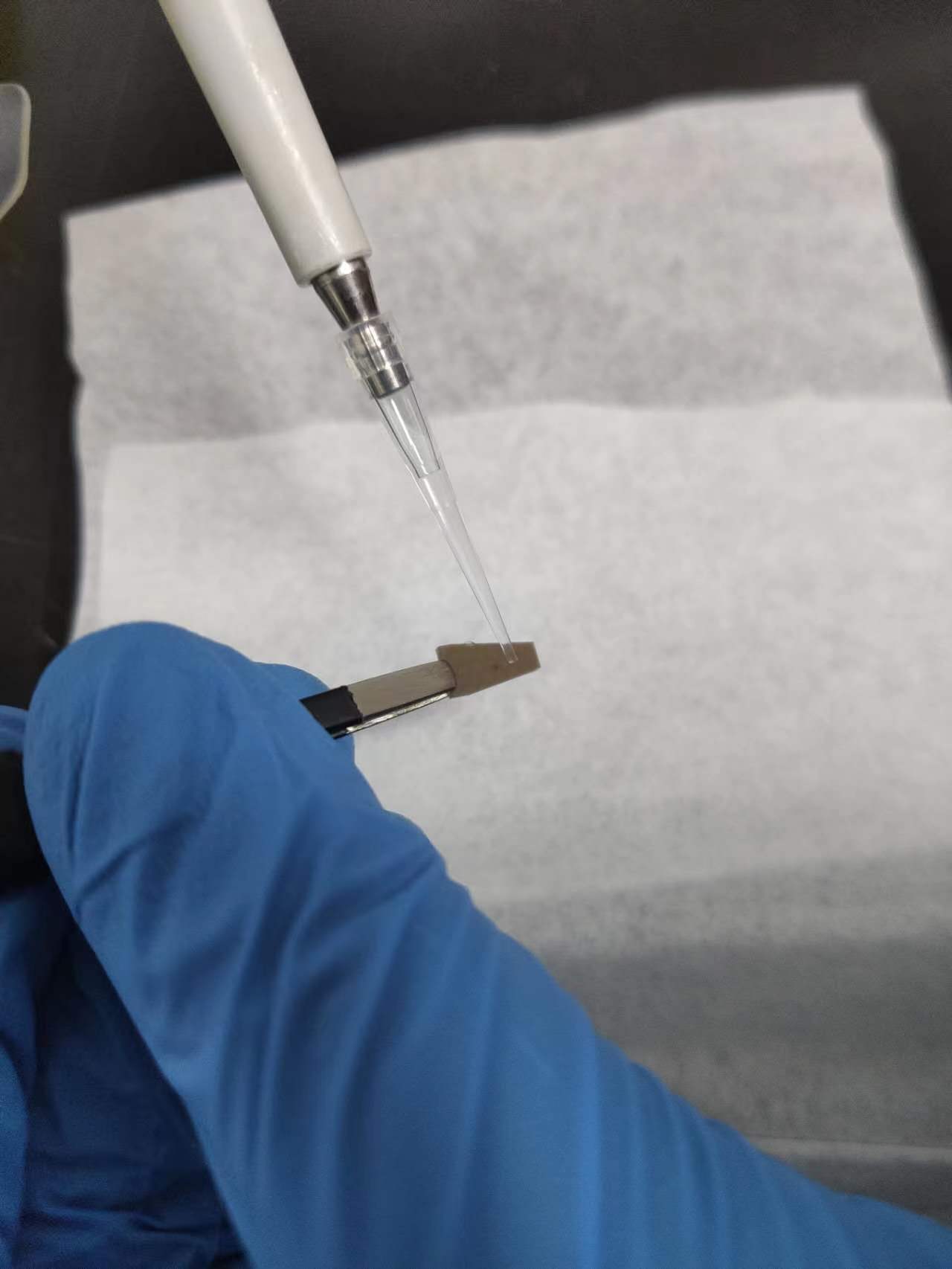


Loading the sample uniformly to cover the surface of the chip

1. After all the liquid was drawn into the chip (5-10 seconds), another 5 µl of loading enzyme mix (0.8 µl of Bsu DNA Polymerase, Large Fragment (catalog no. M0330, NEB); 0.6 µl of RNase H (catalog no. M0297, NEB); 0.6 µl of USER enzyme (catalog no. M5505, NEB); 3 µl of KAPA HiFi HotStart ReadyMix) was added into the chip.
2. Put the chip back into the tube, make sure the chip was stuck in the tube to avoid the floating of chip after adding the oil.
3. Add 150 µl of sealing oil (catalog no. 12005, JN Medsys) carefully along the tube wall to seal the chip.


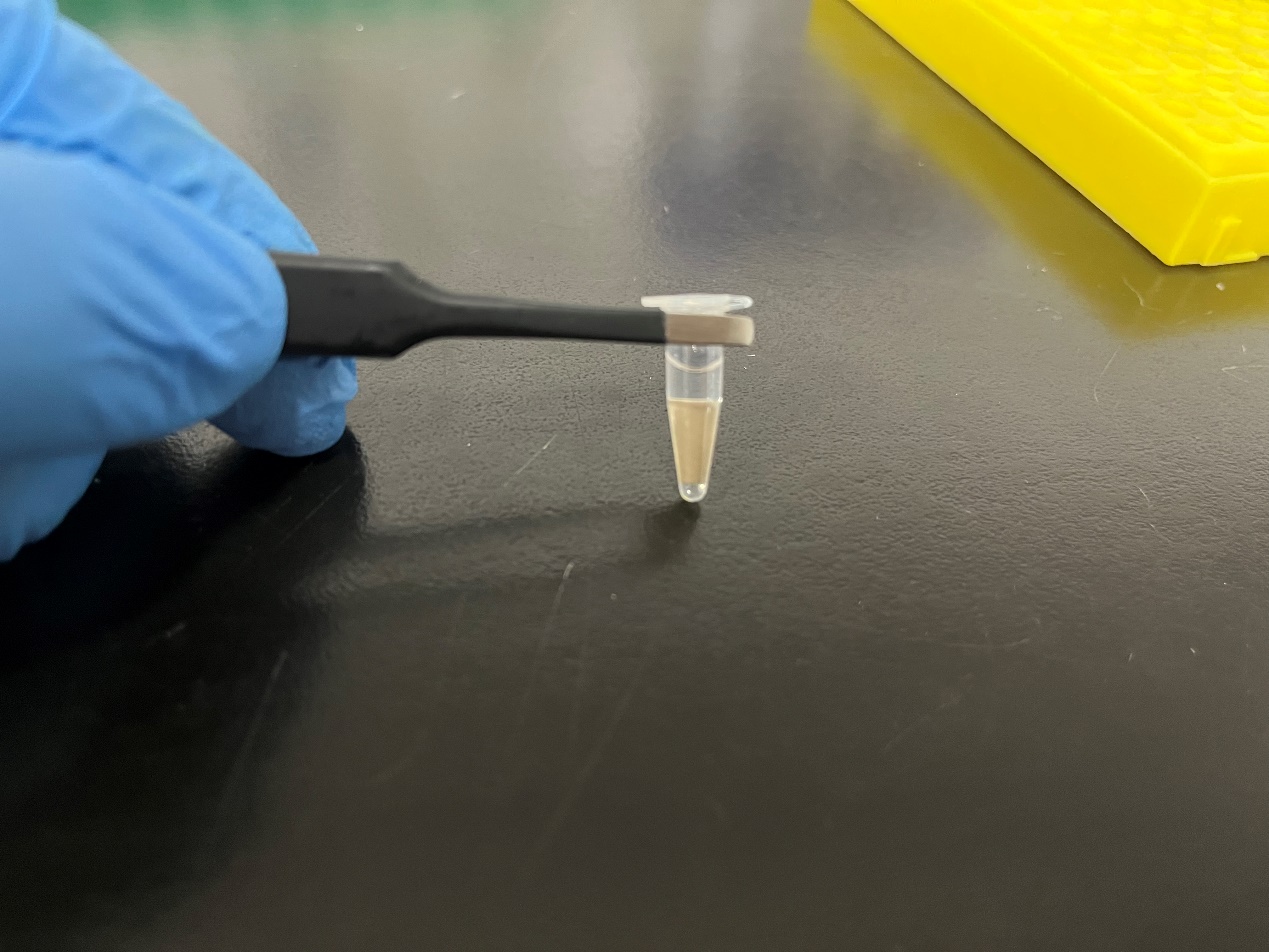


Oil sealing

**Synthesis of template strand and linear amplification in one step reaction**

1. Cover the lid and put the PCR tubes strip in a thermocycler. The reaction was performed as follows:

37 °C for 1 hour

72 °C for 30 min

98 °C for 10 min

10 cycles of:

98 °C for 3 min

55 °C for 3 min

72 °C for 4 min

Final incubation at 72 °C for 30 min

4 °C Hold

**Construction of sequencing library (RNA-seq)**

1. The sealing oil was gently removed after the preamplification step.
2. 80 µl of nuclease-free water with 6× DNA loading dye (catalog no. R0611, Thermo Fisher) was directly added into the chip.
3. The tubes containing the chips were put into a high-speed bench top centrifuge and centrifuged for 5 minutes to collect all the liquid.
4. The liquid with loading dye was separated and transferred to another new PCR tube strip.


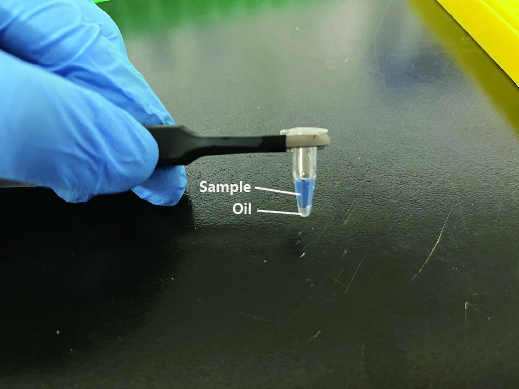


1. The tubes were placed on a magnetic stand to remove all the beads.
2. The preamplification library was purified using 1.5× volume of VANTS DNA cleaning beads (catalog no. N411, Vazyme Biotech).
3. 18 μl of the purified preamplification library was mixed with 1 μl of MGI_P5_primer (Supplementary Table 1, Sangon Biotech), 1 μl of MGI_P7_primer and 20 μl of KAPA HiFi HotStart ReadyMix.
4. Sequencing library PCR was performed as follows:

72 °C for 5 min

98 °C for 3 min

10 cycles of:

98 °C for 20 s

60 °C for 30 s

72 °C for 1 min

Final incubation at 72 °C for 5 min

10 °C Hold

1. The final RNA-seq library was purified using 1.2× VAHTS DNA cleaning beads. The concentration of the library was detected on a Qubit 3.0 (Invitrogen).

***Microwell-seq3 ATAC-seq:***

**Nucleus preparation**

Nucleus preparation protocols for ATAC-seq are the same as in RNA-seq. Fresh nuclei could be resuspended in cold freezing buffer (50 mM Tris-HCl (pH 8.0), 25% glycerin, 5 mM magnesium acetate, 0.1 mM EDTA) and stored at -80℃ for at least a week.

**Tn5 transposase complex assembly**

1. For Tn5 transposome complex assembly, Tn5_primer_A, 384 barcoded Tn5_P5primer_B and Tn5_P7primer_C with MGI adaptor (Supplementary Table 1, Sangon Biotech) were dissolved in TE buffer (10 mM Tris-HCl, 0.1 mM EDTA; pH8.0) to a final concentration of 100 μM.
2. Tn5_primer_A, 384 barcoded Tn5_P5primer_B and Tn5_P7primer_C were mixed at ratio of 2:1:1 in four 96-well plates (8 µl for each well).
3. The plates were placed in thermocyclers and oligos were annealed as follows:

95 °C for 2 min

slow cooling to 20 °C with a temperature ramp of −0.1 °C s−1

1. The annealed mixture was diluted to a final concentration of 1.4 μM in each well.
2. 3.75 µl of Tn5 transposase mix (10 µl of Tn5 (catalog no. S111, Vazyme), 33 μl of coupling buffer (catalog no. S111, Vazyme), 332 μl of dilution buffer (catalog no. S111, Vazyme)) and a 1.25 µl volume containing 1.4 μM concentration of the annealed mixture were added to four 96-well plates.
3. The plates were incubated at 25 °C (room temperature) for 1 hour. The final Tn5 transposase-containing plates were stored at -20 °C.

**Tagmentation**

1. For tagmentation, nuclei were washed with RSBT buffer and counted. Then, 2× TD buffer (20 mM Tris-HCl (pH7.4), 10 mM MgCl2, 20% dimethylformamide (catalog no. D4551, Sigma‒Aldrich)) was prepared and stored at 4 °C.
2. Nuclei were resuspended in tagmentation mix (25 µl for each well: 12.5 µl of 2× TD buffer, 8 µl of 1× PBS, 0.25 µl of 10 % Tween 20, 0.25 µl of 1% digitonin (catalog no. A601152, Sangon Biotech), 2 µl of assembled Tn5, 2 µl of nuclease-free water) and partitioned into four 96-well plates (10,000 nuclei in each well).
3. Tagmentation was performed at 50 °C for 30 minutes. The plates were then placed on ice for 5 minutes to stop the reaction. Nuclei were pooled and washed twice with RSB buffer before loading into the chips.

**Chip-loading (ATAC-seq)**

1. The beads were washed twice with RSB buffer before use. The nuclei and beads were resuspended in loading mix. 20,000 nuclei and 20,000 barcoded beads were loaded into one chip.
2. Loading mix with nuclei and beads: 2 µl of 5× KAPA HiFi Fidelity Buffer (Roche, catalog no. KB2500), 1 µl of 10 mM KAPA dNTP mix, 7 µl of 50 mM EDTA, 4 µl of RSB buffer with nuclei and beads.
3. For experiment with SpikeIn oligos to help the correction of beads data merging after sequencing^[^[^2^](#_ENREF_2)^]^, loading mix with nuclei and beads: 2 µl of 5× KAPA HiFi Fidelity Buffer (Roche, catalog no. KB2500), 1 µl of 10 mM KAPA dNTP mix, 7 µl of 50 mM EDTA, 3.7 µl of RSB buffer with nuclei and beads, 0.3 µl of 100 µM ATAC_SpikeIn_oligos. (Supplementary Table 1)
4. The chip was taken out using a flat tip tweezers. The loading mix with nuclei and beads was pipetted a few times and quickly added to the chip surface using a flat end pipette tip.
5. Put the chip back into the tube and cover the lid. The tubes were placed into a 50 °C incubator for 30 minutes to release the DNA fragments from the nuclei.
6. Another 6.6 µl of loading enzyme mix (2 µl of 5× KAPA HiFi Fidelity Buffer, 1 µl of nuclease-free water, 1 µl of 1U µl-1 KAPA HiFi HotStart DNA Polymerase (catalog no. KE2502, Roche), 2 µl of 400 mM MgCl2, 0.6 µl of USER enzyme) was added into the chip.
7. Put the chip back into the tube, make sure the chip was stuck in the tube to avoid the floating of chip after adding the oil.
8. Add 150 µl of sealing oil carefully along the tube wall to seal the chip.
9. Cover the lid and put the PCR tubes strip in a thermocycler. The reaction was performed as follows:

37 °C for 1 hour

72 °C for 30 min

98 °C for 10 min

10 cycles of:

98 °C for 3 min

55 °C for 3 min

72 °C for 4 min

Final incubation at 72 °C for 30 min

4 °C Hold

**Construction of sequencing library (ATAC-seq)**

1. The sealing oil was gently removed after the preamplification step.
2. 80 µl of nuclease-free water with 6× DNA loading dye was directly added into the chip.
3. The tubes containing the chips were put into a high-speed bench top centrifuge and centrifuged for 5 minutes to collect all the liquid in the chips.
4. The liquid with loading dye was separated and transferred to another new PCR tube strip.
5. The tubes were placed on a magnetic stand to remove all the beads.
6. The preamplification library was purified using 1.5× volume of VANTS DNA cleaning beads.
7. 18 μl of the purified preamplification library was mixed with 1 μl of MGI_P5_primer, 1 μl of MGI_P7_primer and 20 μl of KAPA HiFi HotStart ReadyMix.
8. Sequencing library PCR was performed as follows:

72 °C for 5 min

98 °C for 3 min

10 cycles of:

98 °C for 20 s

60 °C for 30 s

72 °C for 1 min

Final incubation at 72 °C for 5 min

10 °C Hold

1. The final ATAC-seq library was purified using two rounds of size selection: 0.55× VAHTS DNA cleaning beads was used for the first round, and the supernatant was collected; 1.0× VAHTS DNA cleaning beads was used for the second round. The concentration of the library was determined with a Qubit 3.0 (Invitrogen).

**Reference**

1. Han X, Wang R, Zhou Y, Fei L, Sun H, Lai S, Saadatpour A, Zhou Z, Chen H, Ye F, Huang D, Xu Y, Huang W, Jiang M, Jiang X, Mao J, Chen Y, Lu C, Xie J, Fang Q, Wang Y, Yue R, Li T, Huang H, Orkin SH, Yuan GC, Chen M, Guo G. Mapping the Mouse Cell Atlas by Microwell-Seq. **Cell** **2018**, 173(5)**:** 1307.

2. Lareau CA, Duarte FM, Chew JG, Kartha VK, Burkett ZD, Kohlway AS, Pokholok D, Aryee MJ, Steemers FJ, Lebofsky R, Buenrostro JD. Droplet-based combinatorial indexing for massive-scale single-cell chromatin accessibility. **Nat Biotechnol** **2019**, 37(8)**:** 916-+.
